# Insights Into the Oxygen Evolution Reaction on an Mn‐Bearing Hematite Electrocatalyst in an Acidic Electrolyte

**DOI:** 10.1002/cssc.71101

**Published:** 2026-10-04

**Authors:** Moritz Karl Rosenthal, Kim Eklund, Eeva‐Leena Rautama, Lilian Moumaneix, Polina Kalachikova, Nastaran Farrahi, Xiangze Kong, Antti J. Karttunen, Tanja Kallio

**Affiliations:** ^1^ Department of Chemistry and Material Science School of Chemical Engineering Aalto University Espoo Finland

**Keywords:** acidic oxygen evolution reaction, design of experiments, Mn‐doped iron oxide, platinum group metal‐free catalysts, Raman spectroscopy, structural disorder

## Abstract

Platinum group metal‐free catalysts have attracted significant attention due to the high cost and scarcity of iridium for the oxygen evolution reaction (OER) in acidic media. However, the fundamental understanding of catalyst formation and active species under acidic conditions remains limited. In this work, iron–manganese oxide catalysts based on FeA2−xMnAxOA3 were synthesized via a hydrothermal approach followed by calcination. The as‐prepared materials consist of poorly crystalline Fe_2_O_3_/FeOOH and MnO_2_ phases that transform into a disordered mixed‐metal oxide upon thermal treatment. Raman spectroscopy supported by quantum‐chemical calculations indicates the formation of a solid solution in which Mn induces lattice distortion and partial disruption of Fe─O─Fe bonding within the hematite structure. The highest OER activity and stability are obtained after calcination at 350 °C, corresponding to an optimum degree of structural disorder for adsorption processes. Operando Raman spectroscopy reveals potential‐dependent spectral changes, including the emergence of a band at ≈450 cm^−1^ and enhanced intensity at ≈610 cm^−1^, indicating the formation of high‐valent surface species during OER operation. These findings suggest cooperative Fe–Mn interactions and a possible role for substitution‐induced structural disorder in promoting OER activity.

## Graphical abstract

This work investigates Mn incorporation into hematite‐based Fe_1.5_Mn_0.5_O_3_ and its impact on crystal structure and disorder using in situ high‐temperature XRD and quantum chemical calculations. Calcination controlled structural disorder enhances acidic OER activity, while operando Raman spectroscopy provides mechanistic insight into the roles of Fe and Mn during operation.

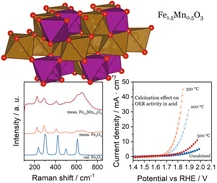

## 1 Introduction

Hydrogen holds great promise as a clean fuel for industrial and transportation applications due to its high specific energy density and the lack of viable alternatives for energy‐intensive processes [1]. However, the majority of hydrogen is still produced through conventional industrial methods that emit substantial amounts of greenhouse gases, limiting progress toward a more sustainable energy system [2]. An alternative is the production of hydrogen via direct water splitting using electrolyzer technologies, including alkaline electrolyzers (AEL), anion exchange membrane electrolyzers (AEMEL), and proton exchange membrane electrolyzers (PEMEL). Due to their high energy efficiency, low Ohmic resistance, and the ability to deliver high‐purity hydrogen, PEMELs are expected to become one of the most mature technologies with an expected capacity deployment of up to 50% in the future [3]. The operation of PEMELs requires efficient electrocatalysts to drive the water‐splitting reactions, namely, the hydrogen evolution reaction (HER) at the cathode and the oxygen evolution reaction (OER) at the anode [4]. The OER remains a significant bottleneck due to its sluggish four‐electron transfer mechanism, typically resulting in high overpotentials (≥1.4 V_RHE_) [5]. Developing effective electrocatalysts for the OER under acidic conditions is particularly challenging, as the reaction occurs at high oxidative potentials (1.6–2.0 V_RHE_), often in combination with elevated temperatures (50–80 °C), which accelerates the degradation of most metal‐based materials [6]. To address these challenges, state‐of‐the‐art OER electrocatalysts typically incorporate IrOA2, RuOA2, or mixed oxides of these platinum group metals (PGMs), which offer a favorable balance between activity and stability under acidic and oxidative conditions [7]. As a result, PGM‐based catalysts have been extensively studied with respect to their synthesis, crystallinity, particle size, and the effects of these structural features on their OER performance, durability, and mechanistic behavior [8]. In contrast, PGMs are extremely scarce and expensive, with more than 80% of the global supply sourced from South Africa, creating a critical dependence that poses risks under geopolitical instability [9]. A recent study by Minke et al. [9] modeled the global demand for iridium through 2070 and concluded that, under scenarios that involve widespread industrial decarbonization and large‐scale electrolyzer deployment for hydrogen production, current Ir supply (maximum ≈ 8 t year^−1^) will only be sufficient if several conditions are met. Specifically, the Ir loading must be reduced from 2.0 to 0.4 mg cm^−2^, together with an increase in the power densities of PEMEL from 3 to 8 W cm^−2^ and a rate of recycling of 90% Ir by 2035 [9]. As a result, numerous studies have focused on the development and evaluation of PGM‐free electrocatalysts for the OER under acidic conditions, incorporating earth‐abundant elements such as Fe, Mn, Sb, and Ni [10, 11]. Despite these efforts, investigations of catalyst formation pathways and fundamental reaction mechanisms remain limited. This lack of mechanistic understanding continues to hinder the rational design, optimization, and broader implementation of PGM‐free OER catalysts [12].

This study investigates hydrothermally grown iron and manganese based oxides, including FeA2OA3 and FeA2−xMnAxOA3, deposited on Ti substrates, followed by calcination in air. Fe and Mn are highly abundant and low‐cost transition metals that have shown promising properties toward acidic OER electrocatalysis, while Mn incorporation into Fe‐based oxides can significantly enhance OER performance [13]. The materials are analyzed using complementary multiscale characterization techniques, including Raman spectroscopy supported by quantum chemical calculations with density functional theory (DFT), X‐ray diffraction (XRD), scanning electron microscopy (SEM), transmission electron microscopy (TEM), energy‐dispersive X‐ray spectroscopy (EDX), and inductively coupled plasma optical emission spectroscopy (ICP‐OES) in order to elucidate their structural evolution and the formation of catalytically active species. The electrochemical performance toward the oxygen evolution reaction (OER) is evaluated under acidic conditions, and the influence of calcination parameters is systematically investigated using a response surface design of experiments (DOE). To further understand the crystallization behavior during the calcination, in situ high‐temperature XRD (HTXRD) is combined with synchrotron wide‐angle X‐ray scattering (WAXS), thermogravimetric analysis (TGA), differential scanning calorimetry (DSC), and mass spectrometry (MS). Finally, operando Raman spectroscopy is employed to probe potential‐dependent surface transformations during the OER and to provide mechanistic insights into the role of Fe and Mn under anodic conditions.

## 2 Results and Discussion

### 2.1 Electrochemical Characterization

Before discussing the electrochemical results, it is important to note that the optimization of composition, hydrothermal synthesis temperature, and hydrothermal reaction time was systematically investigated in a previous work [13]. That study demonstrated that pure Fe‐ or Mn‐oxide films grown on Ti substrates exhibit negligible OER activity. Therefore, the formation of mixed‐metal oxide phases of the type FeA2−xMnAxOA3 is required to obtain catalytically active materials. The present section discusses the electrochemical behavior of mixed iron–manganese oxide phases as a function of calcination temperature (between ≈209 and ≈491 °C) and calcination duration (0.6 and 3.4 h) in air using a response surface design of experiments (DOE) with more experimental details shown in Figure 1a or Table S4. The materials were labeled according to the notation Fe_a_Mn_b_T_c_t_d_Ti_F/M_, where *a* and *b* represent the nominal introduced Fe and Mn fractions, *T*_c_ denotes the calcination temperature (in ‍°C), *t*_d_ the calcination duration (in h), and Ti_F_ and Ti_M_ indicate whether Ti foil or Ti mesh was used as the substrate.

**FIGURE 1:**
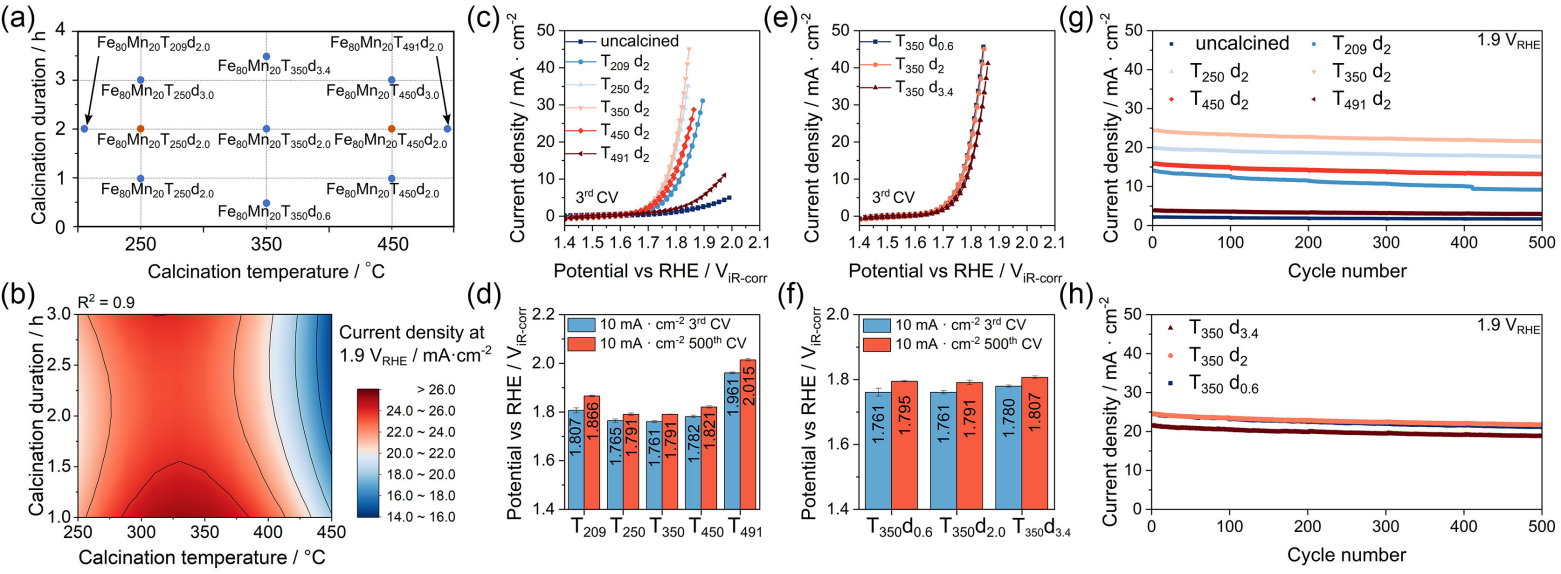
OER activity and stability measurements of the catalysts grown on the Ti mesh, recorded in a three‐electrode setup using a reversible hydrogen electrode (RHE) reference and a Pt wire counter electrode in 0.1 M HClOA4 at 25 °C. (a) Experimental calcination conditions investigated within the response surface design of experiments (DOE) and additional measurements. (b) DOE results derived from CV measurements at 1.9 V versus RHE (current density response surface, uncompensated). (c) iR‐corrected CVs recorded between 1.4 and 2.0 V versus RHE at a scan rate of 10 mV s^−1^ for electrodes calcined at different temperatures. (d) Corresponding potentials required to reach a current density of 10 mA cm^−2^ in the 3rd and 500th CV cycles. (e) iR‐corrected CVs recorded between 1.4 and 2.0 V versus RHE at a scan rate of 10 mV s^−1^ for electrodes calcined at 350 °C with varying calcination durations. (f) Corresponding potentials required to reach a current density of 10 mA cm^−2^ in the 3rd and 500th CV cycles. (g) Current densities recorded at 1.9 V versus RHE (uncompensated) over 500 cycles between 1.4 and 2.0 V versus RHE at a scan rate of 100 mV s^−1^ for electrodes calcined at different temperatures. (h) Current densities recorded at 1.9 V versus RHE (uncompensated) over 500 cycles between 1.4 and 2.0 V versus RHE at a scan rate of 100 mV s^−1^ for electrodes calcined at 350 °C with varying calcination durations.

#### 2.1.1 Influence of Calcination Conditions on the Catalytic OER Activity

The cyclic voltammograms (CVs) of all calcined electrodes show an expected sharp increase in current density after the onset at approximately 1.7–1.8 V_RHE_, which is attributed to the OER. Among the calcination temperatures investigated, the DOE results reveal that the current density is influenced primarily by the calcination temperature, with a projected optimum around 330 °C (Figure 1b). Cyclic voltammetry shows that the uncalcined sample (${\mathrm{Fe}}_{80}{\mathrm{Mn}}_{20}{\mathrm{Ti}}_{M}$) exhibits negligible OER activity (Figure 1c). The highest performance is achieved after electrode calcination between 250 and 350 °C, both reaching 10 mA cm^−2^ at ≈1.76 V_iR−corr_ versus RHE (Figure 1d). Temperatures outside this range result in significantly reduced activity, as observed for the samples calcined at 209  °C (≈1.81 V_iR−corr_ vs. RHE) and 491 °C (≈1.96 V_iR−corr_ vs. RHE). Although onset potentials for the 209 and 491 °C samples are relatively high (1.70 and 1.78 V_iR−corr_ vs. RHE), the remaining catalysts exhibit similar onset potentials near 1.64  V_iR−corr_ vs. RHE. Mass‐normalized OER activities are shown in Figure S1. The activity trend remains unchanged after normalization by catalyst loading, indicating that the observed performance differences originate from intrinsic catalyst properties rather than variations in deposited catalyst mass. The catalyst calcined at 350 °C exhibits the highest mass activity, reaching approximately 100 mA mg^−1^ at ≈1.84 V_iR−corr_ versus RHE.

Electrochemical impedance spectroscopy (EIS) analysis and equivalent circuit modeling (Figure S2) support these findings. Both the charge–transfer resistance (*R*_ct_) and adsorption resistance (*R*_a_) are substantially higher for the uncalcined sample and the samples calcined at 491 °C (Figure S3). Notably, the difference in *R*_a_ is much more pronounced than in *R*_ct_, suggesting that adsorption processes are the dominant factor limiting the reaction [13]. The electrochemical surface area (as reflected in the double‐layer capacitance, *C*_dl_) decreases with increasing calcination temperature from 209 to 491 °C. The observed decrease in double‐layer capacitance may originate from several factors, including thermally induced particle coarsening, increased crystallinity, and changes in porosity (Figures S3 and S4) [14]. In contrast, the adsorption capacitance (*C*_a_) reaches its highest values at 250 and 350 °C, suggesting that these temperatures produce the most favorable surface structure for adsorption processes [13].

The influence of calcination duration is more subtle and should be interpreted with caution due to the small variations observed between samples. Although electrodes calcined at identical temperatures (250, 350, 450 °C) show comparable OER activities, extended calcination time generally leads to a slight decrease in performance (Figure 1e). At 350 °C, a short treatment time of 0.6 h produces activity comparable to that obtained after 2 h, whereas increasing the duration to 3.4 h results in reduced activity (Figure 1f).

EIS data reinforce these results (Figure S3). While all 350 °C samples exhibit similar *R*_ct_ and *R*_a_, the adsorption capacitance (*C*_a_) is highest for shorter calcination durations (0.6 and 2.0 h), again highlighting the central role of adsorption processes in determining catalytic activity. The 2 h sample, in particular, combines the highest *C*_a_ with the lowest *R*_a_, identifying it as the optimal treatment.

#### 2.1.2 Influence of Calcination Conditions on the Catalytic OER Stability

The ASTs (CV cycling between 1.4 and 2.0  V_RHE_) reveal broadly similar stability behavior across all calcination temperatures, with a current loss of approximately 10%–20% over 500 cycles (Figure 1g). The samples calcined at 250 and 350 °C exhibit the most favorable stability (≈90% ± 2% retention) among all compositions. In contrast, electrodes calcined at both lower temperatures (209 °C; ≈67±2% retention) and higher temperatures (450 °C; ≈83% ± 1% retention) show a faster decrease in activity. The stability of electrodes calcined for 0.6 h (≈90% ± 3%) and 3.4 h (≈89% ± 1%) is comparable to that of the 2 h sample within the tested cycling duration (Figures 1h and S5). Considering the CV measurement at the end of the test, electrodes calcined at 350 °C for 2 h provide the best balance between high OER activity and sustained electrochemical stability and are therefore identified as the optimal calcination temperature (Figure S5).

The stability evaluation presented here was initially limited to 500 accelerated stability cycles to verify that the calcination‐optimized catalysts follow the same stability trend as previously reported [13]. Additional stability measurements were performed at 50 mA cm^−2^ for the optimized sample calcined at 350 °C for 2 h, with the results shown in the Figure S6. The measurement shows a progressive increase in the iR‐corrected potential from 1.85 to 2.10 V_RHE_ required to maintain 50 mA cm^−2^ during the 77 h (≈3.3 mV  h^−1^) measurement (Figure S6). The measured potential is also sensitive to continuous electrolyte consumption and evaporation, which was compensated by periodic electrolyte replenishment and resulted in small but distinct potential drops. Notably, the potential increase became progressively faster during the measurement, with an increase of approximately 1 mV h^−1^ during the first 30 h, ≥3 mV  h^−1^ between 40 and 60 h, and ≥6 mV h^−1^ during the final stage of the testing. This behavior indicates progressively increasing loss of electrochemical performance during prolonged operation and is also reflected in the polarization curves recorded throughout the measurement (Figure S6).

EIS analysis provides further insight into the evolution of the electrode during extended operation. The Nyquist (Figure S6) and Bode plots (Figure S6) indicate a progressive increase in the charge–transfer resistance accompanied by a decrease in the adsorption resistance. This evolution is supported by the equivalent‐circuit modeling, with *R*_ct_ increasing from 0.9 ± 0.1 to 6.5 ± 0.1 Ω over the 70 h testing period. In contrast, *R*_a_ decreases from 0.9 ± 0.1 to 0.1 ± 0.1 Ω, with substantially smaller changes observed after approximately 20 h of testing. The different evolution of *R*_ct_ and *R*_a_ coincides with the change in the potential evolution observed during extended operation. While *C*_dl_ remains relatively constant throughout the testing period, suggesting no substantial change in the apparent electrochemically accessible surface area, *R*_a_ progressively decreases alongside with increasing *C*_a_. These trends are consistent with observations from our previous study [13].

#### 2.1.3 Activity–Stability Comparison

The activity and stability of representative PGM‐free OER catalysts in acidic environment are summarized in Table S1, while more comprehensive overviews are available in recent review articles [15, 16, 17, 18, 19]. In general, Co‐based mixed‐metal oxides exhibit the highest reported activities (often ≤400 mV at 10 mA cm^−2^) and longest reported operating times, ranging from hundreds to thousands of hours, among PGM‐free acidic OER catalysts. However, cobalt is considered a critical raw material and was therefore not investigated in the present study [20]. The optimized FeA1.5MnA0.5OA3 catalyst exhibits an overpotential of approximately 530 mV at 10 mA cm^−2^, placing it within the range reported for other Fe‐ and Mn‐based oxide catalysts, which typically require overpotentials of 550–670 mV under comparable conditions [10, 21, 22, 23]. The optimized catalyst also maintains operation at 50 mA cm^−2^ for 77 h during extended chronoamperometric testing, although the potential increases by approximately 250 mV over the testing period. While direct comparison of stability data is complicated by differences in testing conditions, the observed durability is within the range reported for other PGM‐free acidic OER catalysts in acidic media (Table S1).

### 2.2 Physicochemical Characterization

Both the calcination procedure and Mn substitution within the FeA2OA3 hematite matrix have a significant influence on the catalytic performance of the investigated materials. To distinguish the individual contributions of these two parameters, a series of complementary characterization techniques was employed, combining quantum chemical calculations for Raman spectra modeling with experimental Raman spectroscopy, as well as SEM‐EDX, TEM‐EDX, in situ HTXRD, and TGA‐DSC‐MS. Analysis of the catalyst (FeA1.5MnA0.5OA3) was conducted against pure hematite (FeA2OA3) and a corresponding uncalcined Fe–Mn precursor to enable a systematic comparison of the effects of Mn incorporation.

#### 2.2.1 Raman Spectroscopy

The crystal structure of *α*‐Fe_2_O_3_ and two ordered models of FeA1.5MnA0.5OA3 with different Mn substitution configurations within the hematite lattice, used for the quantum‐chemical calculations, are shown in Figure 2. The measured Raman spectrum of the iron oxide based ${\mathrm{Fe}}_{100}{\mathrm{Mn}}_{0}T_{350}d_{2}{\mathrm{Ti}}_{F}$ (referred to as FeA2OA3) exhibits the characteristic vibrational modes of pure hematite phase (Figure 3a), with bands at approximately 223 cm^−1^ (*A*_1g_), 245 cm^−1^ (*E*_g_), 291 cm^−1^ (*E*_g_), 406 cm^−1^ (*E*_g_), 498 cm^−1^ (*A*_1g_), and 610 cm^−1^ (*E*_g_), in agreement with literature values [25, 26, 27] and the DFT calculations (Figure 3b). Interestingly, a weak longitudinal optical (LO) band at 660 cm^−1^ (*E*_u_), along with a second‐order overtone (2LO) around 1321 cm^−1^, is also observed. A previous study has shown that the intense 2LO feature can be reproduced using a sophisticated computational approach that combines the electronic dispersion curve of a polaron distorted lattice with the vibrational dispersion curve of bulk hematite [28]. These features have been attributed to various origins by the existing experimental literature. The modes, which are not observed in the standard DFT calculations used here (Figure S7), have been previously reported to originate from the presence of impurity iron oxides such as maghemite (*γ*‐Fe_2_O_3_) or magnetite (FeA3OA4) structures with main peaks around 670 cm^−1^ [27, 29]. The absence of additional peaks attributable to these phases suggests that the 1LO 660 cm^−1^ band and its 2LO overtone more likely originate from structural disorder in the hematite lattice. These findings are in agreement with previous experimental works reporting both bands around 660 and 1321 cm^−1^ for pure *α*‐Fe_2_O_3_ [27, 30].

**FIGURE 2:**
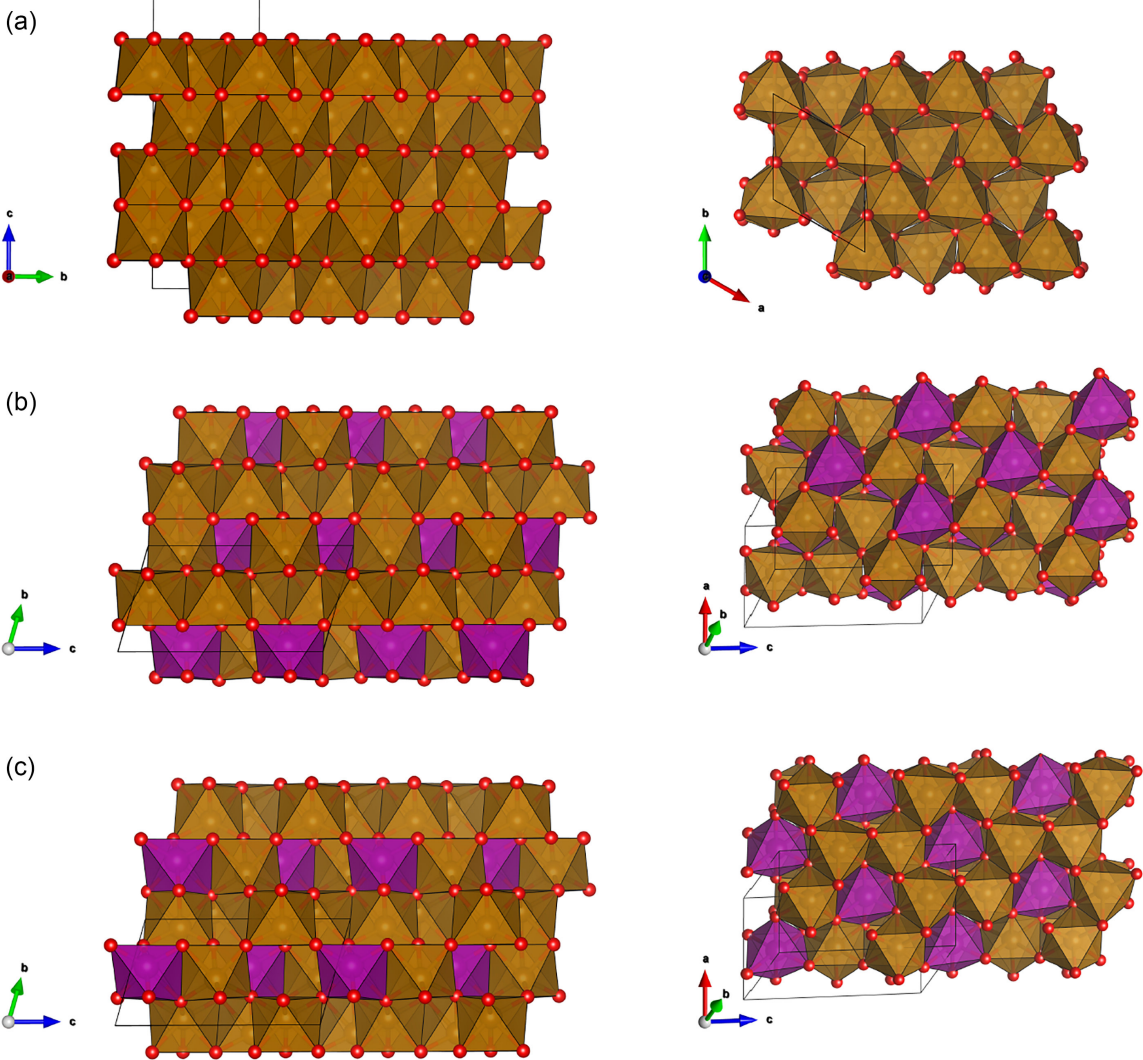
Structural models used for quantum chemical calculations: (a) *α*‐Fe_2_O_3_ ($R\bar{3}c$), (b) a‐Fe_1.5_Mn_0.5_O_3_ (*P*1), and (c) b‐Fe_1.5_Mn_0.5_O_3_ (*P*1) representing two distinct Mn substitution configurations within the distorted hematite lattice. Fe atoms and FeO_6_ coordination octahedra are shown in brown, Mn atoms and MnO_6_ coordination octahedra in purple, and O atoms in red. The black lines show the unit cell of each structure. Structures were visualized using VESTA [24].

**FIGURE 3:**
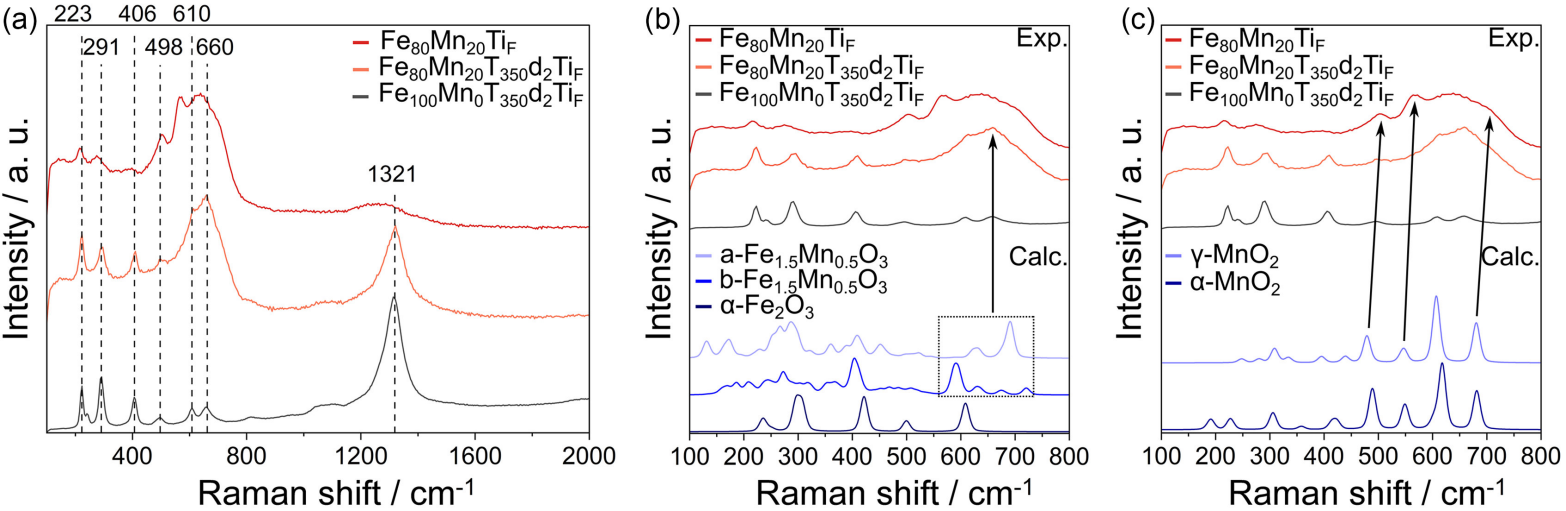
(a) Raman spectra of ${\mathrm{Fe}}_{100}{\mathrm{Mn}}_{0}T_{350}d_{2}{\mathrm{Ti}}_{F}$, ${\mathrm{Fe}}_{80}{\mathrm{Mn}}_{20}T_{350}d_{2}{\mathrm{Ti}}_{F}$, and uncalcined ${\mathrm{Fe}}_{80}{\mathrm{Mn}}_{20}{\mathrm{Ti}}_{F}$ recorded between 100 and 2000 cm^−1^ using a 532 nm excitation wavelength at 5% laser power. Peak positions are indicated at the top of the figure. (b) Corresponding Raman spectra between 100 and 800 cm^−1^ together with DFT‐calculated Raman spectra for *α*‐Fe_2_O_3_, a‐Fe_1.5_Mn_0.5_O_3_, and b‐Fe_1.5_Mn_0.5_O_3_ highlighting the spectral signatures associated with Mn substitution and lattice distortion. (c) Comparison of the experimental Raman spectra with DFT‐calculated Raman spectra of *α*‐ and *γ*‐MnO_2_.

The measured spectrum of ${\mathrm{Fe}}_{80}{\mathrm{Mn}}_{20}T_{350}d_{2}{\mathrm{Ti}}_{F}$ (referred to as FeA1.5MnA0.5OA3) closely resembles that of FeA2OA3, suggesting retention of the hematite structure after partial substitution of Mn, in agreement with the peak positions calculated for the material FeA1.5MnA0.5OA3 (Figure 3a,b). However, all observed Raman bands show peak broadening upon Mn introduction. In particular, contributions between 610 and 660 cm^−1^ increase in intensity and broaden significantly compared with the pure hematite sample. While the presence of manganese oxide phases in the material is possible, the peak positions of various MnOA2 polymorphs (*α*, *β*, and *γ*) [31] are unable to explain the intensity and broadening observed between 610 and 660 cm^−1^ (Figures 3c and S7). Therefore, the reason behind this is likely the formation of the solid solution, which is also supported by the nearly identical ground state energies of the calculated models (a‐Fe_1.5_Mn_0.5_O_3_ and b‐Fe_1.5_Mn_0.5_O_3_) (Figure 2). Inducing Mn into the hematite causes the disruption of the Fe–O–Fe lattice and thus enhances the visibility of weak or symmetry‐forbidden modes [32, 33]. The resulting increased disorder is then evidenced as more intense bands in the spectrum. The Mn‐induced distortion of the original hematite structure is enhanced by the Jahn–Teller effect on the MnA3+ cation (*d* ^4^) [34]. This Jahn–Teller distortion is evident from the increased differences in the Mn─O bond lengths between the two calculated structural models (Table S2). The longest Mn─O bond length becomes 2.28 Å, while nearby Fe─O bonds are also elongated up to 2.25 Å. The normal Fe─O bond length in the calculated model for FeA2OA3 is 2.12 Å. Therefore, the catalyst FeA1.5MnA0.5OA3 is more likely to consist predominantly of a hematite phase with only low levels of Mn‐ or Fe‐based structural impurities.

The spectrum of the uncalcined sample ${\mathrm{Fe}}_{80}{\mathrm{Mn}}_{20}{\mathrm{Ti}}_{F}$ mostly exhibits bands close to those of hematite (*α*‐Fe_2_O_3_), although the bands show less intensity and are slightly shifted, suggesting that an iron oxide or hydroxide phase is already present (Figure ‍3a). However, the feature around 1321 cm^−1^ observed for both *α*‐Fe_2_O_3_ and FeA1.5MnA0.5OA3 is significantly reduced in intensity, indicating that the material exhibits significant differences in structural ordering and/or crystallinity. The additional bands at approximately 502, 564, and 712 cm^−1^ match those of the DFT calculated peak positions of *α*‐MnO_2_ or *γ*‐MnO_2_ (Figures 3c and S7) and a previous study on MnOA2 [31]. This indicates that, after hydrothermal synthesis, the material already consists of poorly crystalline or disordered iron oxide/oxyhydroxide mixed with *α*‐MnO_2_ or *γ*‐MnO_2_ (Figure 3c). Upon calcination at elevated temperatures, these components gradually transform into a more homogeneous mixed‐metal oxide with a hematite‐type structure, forming FeA2−xMnAxOA3. This interpretation is consistent with the reported thermal stability of the MnOA2 polymorphs. δ‐MnO_2_ is commonly obtained as a decomposition product of KMnOA4 (precursor), but is unstable, forming *α*‐MnO_2_ during the hydrothermal synthesis due to elevated temperature and pressure [35]. Upon heating in air, *α*‐MnO_2_ typically transforms into MnA3OA4 and converts into MnA2OA3 at higher temperatures [35], here forming a solid solution with iron oxide into combined FeA1.5MnA0.5OA3.

Additional Raman analysis show similar growth on both Ti mesh and foil substrates. An increase of calcination temperature (450 °C) results in notably sharpened band feature with less intensity around 660 cm^−1^, indicating an increase in structural order of the Mn‐bearing hematite phase (Figure S8). This observation is particularly relevant, as lattice disorder in transition‐metal oxides can modify the local electronic structure and adsorption behavior of oxygenated intermediates, thereby strongly influencing OER electrocatalytic performance [36, 37].

#### 2.2.2 Nanostructure Analysis by TEM

TEM images indicate pronounced morphological differences between the pure Fe sample and the Mn‐containing hematite (Figure 4a–c). Simultaneously, the high‐magnification TEM images reveal lattice spacings of 0.27 nm (104) and 0.38 nm (012), originating from the hematite lattice in all samples [38]. Selected area electron diffraction (SAED) further corroborates the presence of the characteristic hematite planes in both FeA2OA3 and FeA1.5MnA0.5OA3. However, the uncalcined Fe–Mn material shows a less complete pattern with missing reflection rings 012 and 113, suggesting disorder or incompletely formed hematite structure, aligning with the Raman results (Figure 4c). Previous studies investigating hematite hydrothermal growth report that short reaction durations lead to broad XRD reflections, small crystallites, and incomplete hematite lattice ordering and the presence of *α*‐FeOOH [39, 40]. Other works similarly highlight the importance of elevated calcination temperatures and prolonged durations for complete hematite phase formation [41, 42]. These findings underscore that post‐synthesis calcination plays a key role in determining the structural integrity of the catalyst, thereby influencing its intrinsic OER activity.

**FIGURE 4:**
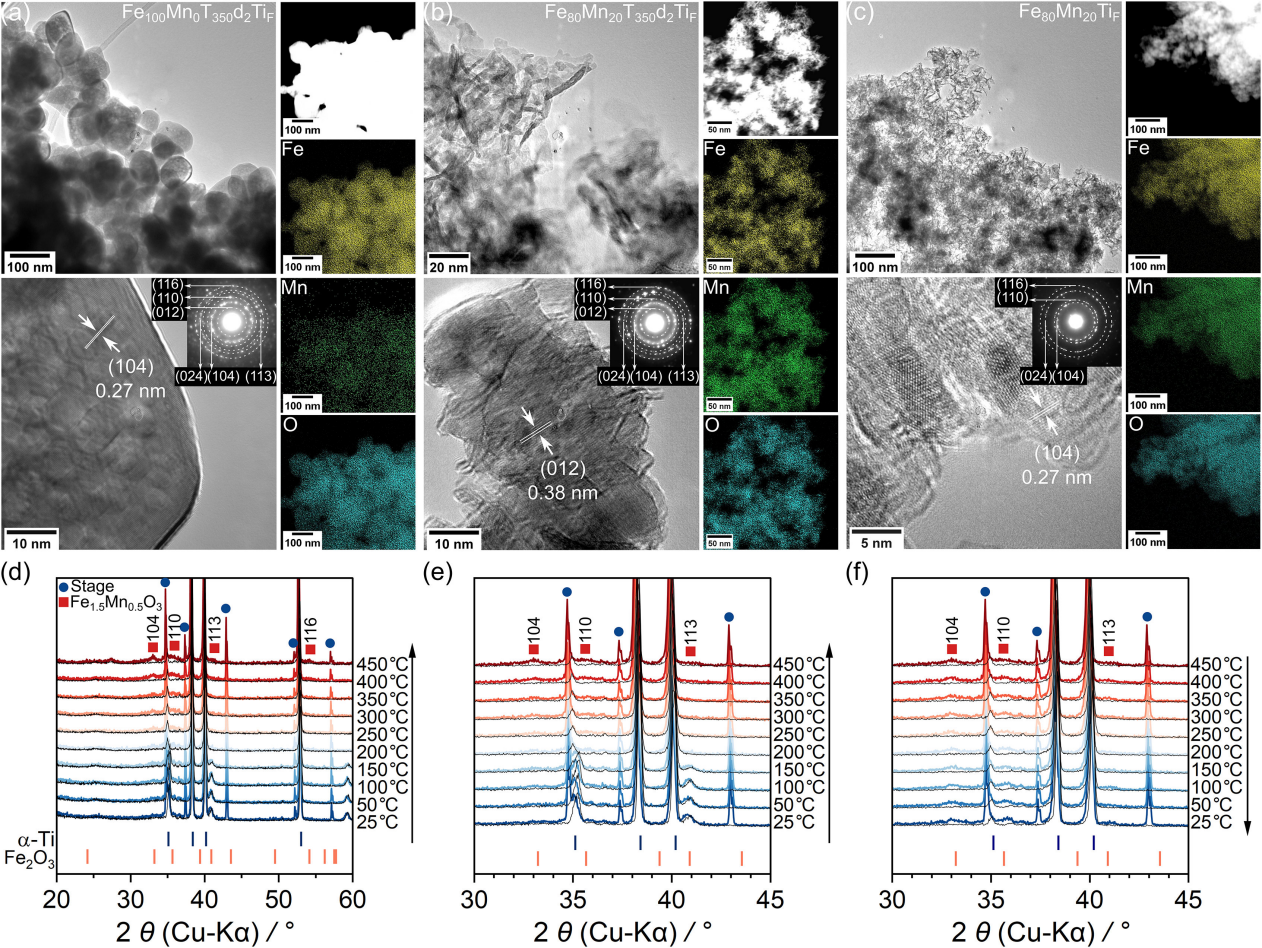
TEM micrographs, selected‐area electron diffraction (SAED) patterns, and elemental mapping of mechanically extracted catalyst layers from Ti foil electrodes acquired at an accelerating voltage of 200 kV: (a) ${\mathrm{Fe}}_{100}{\mathrm{Mn}}_{0}T_{350}d_{2}{\mathrm{Ti}}_{F}$, (b) ${\mathrm{Fe}}_{80}{\mathrm{Mn}}_{20}T_{350}d_{2}{\mathrm{Ti}}_{F}$, and (c) ${\mathrm{Fe}}_{80}{\mathrm{Mn}}_{20}{\mathrm{Ti}}_{F}$. In situ HTXRD patterns of ${\mathrm{Fe}}_{80}{\mathrm{Mn}}_{20}{\mathrm{Ti}}_{F}$ (colored) and the Ti foil substrate (black) recorded in 50 °C increments during controlled heating: (d) 2*θ* = 20°–60° and (e) 2*θ* = 30°–45°. (f) Corresponding in situ HTXRD patterns recorded during controlled cooling in the range 2*θ* = 30°–45°. For clarity, only selected 2*θ* regions are shown.

#### 2.2.3 Catalysts Morphology by SEM and Cross‐Sectional SEM

Performed SEM measurements of FeA2OA3, FeA1.5MnA0.5OA3, and the uncalcined material confirm the morphological changes previously observed by TEM, while also showing complete surface coverage of the Ti foil electrodes by a uniform catalyst layer (Figure 5). FeA2OA3 exhibits relatively large particles (190 ± 30 nm), while all Mn‐containing catalysts show a significant reduction in particle size to ≤100 nm, possibly related to the lattice distortion through Mn substitution (Figure 5). After calcination at 350 °C for 2 h, the initially dispersed nanoparticles appear to merge, forming a more continuous layer on the Ti foil, possibly indicating partial intergrowth with the substrate surface [13]. Additional SEM measurements of the catalysts grown on the Ti mesh show similar growth behavior compared with that observed on the Ti foil (Figure S9). Measurements of the apparent particle diameters from SEM micrographs of the uncalcined electrode and electrodes calcined at 350 and 491 °C reveal an increase from 41 ± 8 to 45 ± 8 and 70 ± 15 nm, respectively (Figure S10). These observations support thermally induced particle coarsening during calcination while suggesting that additional factors, such as increased crystallinity and changes in porosity, also contribute to the reduction in electrochemically accessible surface area. Cross‐sectional SEM images confirm that the Ti mesh fibers are embedded in a catalyst layer with a thickness of approximately 3–5 μm (Figure S11).

**FIGURE 5:**
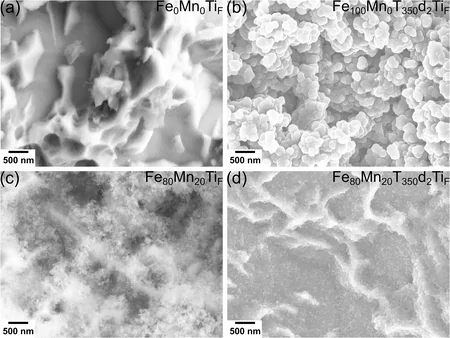
SEM images recorded at an accelerating voltage of 8 kV and a magnification of 20 k of (a) bare Ti foil, (b) ${\mathrm{Fe}}_{100}{\mathrm{Mn}}_{0}T_{350}d_{2}{\mathrm{Ti}}_{F}$, (c) uncalcined ${\mathrm{Fe}}_{80}{\mathrm{Mn}}_{20}{\mathrm{Ti}}_{F}$, and (d) ${\mathrm{Fe}}_{80}{\mathrm{Mn}}_{20}T_{350}d_{2}{\mathrm{Ti}}_{F}$, illustrating the influence of Mn incorporation and calcination on the surface morphology of the catalyst layers.

#### 2.2.4 Semiquantitative and Quantitative Composition by EDX and ICP‐OES

Semiquantitative SEM‐EDX analysis of FeA2OA3 grown on the Ti foil shows only the presence of Fe, O, and Ti, whereas the Fe–Mn samples (${\mathrm{Fe}}_{80}{\mathrm{Mn}}_{20}T_{350}d_{2}{\mathrm{Ti}}_{F}$) grown on the Ti foil exhibit a homogeneous distribution of Fe (78 ± 2 at.%) and Mn (22 ± 2 at.%). This indicates the formation of a compositionally uniform FeA2−xMnAxOA3 phase with *x* ≈ 0.45 (Table S3). For the uncalcined catalyst (${\mathrm{Fe}}_{80}{\mathrm{Mn}}_{20}{\mathrm{Ti}}_{F}$), EDX analysis reveals similar Fe (79 ± 7 at.%) and Mn (21 ± 3 at.%) surface concentrations compared with the calcined electrode (Table S3).

SEM‐EDX analyzes were also performed for all calcined catalysts synthesized on the Ti mesh, yielding Fe and Mn contents in the ranges of 71–81 and 21–29 at.%, respectively. These results indicate reproducible catalyst growth across all electrochemically tested electrodes, with compositions varying within a narrow range of FeA1.4MnA0.6OA3 to FeA1.6MnA0.4OA3, consistent with an average composition close to FeA1.55MnA0.45OA3 (Table S3).

TEM‐EDX mapping reveals a homogeneous distribution of Fe and O within the FeA2OA3 particles, and of Fe, Mn, and O within both FeA1.5MnA0.5OA3 and the uncalcined material (Figure 4a–c). Semi‐quantitative analysis suggests a possible surface enrichment of Mn for the calcined electrode (Fe:Mn ratio of approximately 0.9), corresponding to a nearly equal Fe:Mn surface composition that can be approximated as FeA1.0MnA1.0OA3. This observation is consistent with findings from a previous work (Table S4) [13].

Quantitative ICP‐OES analysis of the powder sample yields an Fe:Mn ratio of approximately 3:1, corresponding to a bulk composition close to FeA1.5MnA0.5OA3. The differences between SEM‐EDX, TEM‐EDX, and ICP‐OES reflect the different sampling volumes and quantification principles of these techniques. While SEM‐EDX and TEM‐EDX probe localized regions of the heterogeneous catalyst, ICP‐OES provides the average bulk composition after complete dissolution. Furthermore, EDX quantification is influenced by factors such as matrix effects, particle morphology, and interaction volume, whereas these effects are largely eliminated in ICP‐OES measurements [43]. Consequently, local compositional variations observed by EDX should not be interpreted as changes in the overall catalyst stoichiometry. Overall, the complementary analyses indicate an Fe‐rich catalyst with a bulk Fe:Mn ratio close to the intended composition, while TEM‐EDX suggests a possible tendency toward Mn enrichment in the catalyst material after calcination (Figure S12).

#### 2.2.5 In Situ High‐Temperature XRD and Synchrotron WAXS

The initial in situ HTXRD patterns of a pure Ti foil and ${\mathrm{Fe}}_{80}{\mathrm{Mn}}_{20}{\mathrm{Ti}}_{F}$ show only minor differences, indicating that the uncalcined Fe–Mn material deposited on the Ti foil is poorly crystalline after hydrothermal synthesis, in agreement with the previous discussion (Figure 4d–f). All patterns for both ${\mathrm{Fe}}_{80}{\mathrm{Mn}}_{20}{\mathrm{Ti}}_{F}$ and the bare Ti foil exhibit the characteristic reflections of *α*‐Ti, with no observable changes in the support material during the heating and cooling cycles. Several additional XRD reflections appear that match those recorded from the stage without any sample present (Figure S13). These artifacts likely result from sample displacement or movement during heating and are therefore not discussed further.

Upon heating, the Ti foil and ${\mathrm{Fe}}_{80}{\mathrm{Mn}}_{20}{\mathrm{Ti}}_{F}$ show the disappearance of certain reflections, for instance, at 2*θ* values of 35.3°, 40.9°, and 59.4°, which may originate from secondary Ti phases or impurities and are thus not of interest. The onset of hematite phase formation occurs between 200 and 250 °C, evidenced by the appearance of broad reflections corresponding to the (104) and (110) planes. The broadness of these peaks indicates the presence of small crystallites, incomplete crystallization, or structural disorder, consistent with earlier observations. Additional characteristic hematite reflections become visible only at temperatures around 450 °C, suggesting that a higher degree of crystallinity requires increased thermal energy. During cooling, the reflection intensities increase but remain broad, confirming small crystallite size and/or disorder‐rich structures. Both samples also show a reflection near 2*θ* = 27°, which may correspond to the formation of a TiOA2 rutile phase [13].

These observations are further supported by the fact that FeA2OA3 exhibits well‐defined reflections characteristic of the hematite phase, whereas the uncalcined material does not show any hematite reflections. Upon calcination, the same sample displays reflections at similar 2*θ* positions, but with significantly lower intensity and broader peak shapes (Figure S14). In situ HTXRD measurements of the material grown on Ti mesh reveal similar behavior to the one grown on Ti foil (Figure S15). A more detailed discussion is provided in the Supporting Information.

Synchrotron wide‐angle X‐ray scattering (WAXS) measurements of FeA2OA3 and FeA1.5MnA0.5OA3 confirm the formation of a phase‐pure hematite‐based *α*‐Fe_2_O_3_ structure ($R\bar{3}c$), in addition to the dominant reflections originating from the *α*‐Ti substrate ($P6_{3}/mmc$) (Figure S16). Consistent with the laboratory XRD measurements, the Fe–Mn mixed oxide exhibits pronounced reflection broadening compared with the pure Fe oxide, indicating increased structural disorder and reduced crystallite coherence lengths. Le Bail‐type profile fitting of the pure Fe oxide yielded lattice parameters of *a* = *b* = 5.0361(2) and *c* = 13.761(1) Å, in good agreement with reported literature values for hematite [44]. For the Mn‐containing sample, the fitting procedure had to be partially constrained due to the strong reflection broadening. Nevertheless, distinct changes in the lattice parameters are observed, including an approximately 1% contraction of the *a* and *b* parameters to 4.988(2) Å and a 0.5% expansion of the *c* parameter to 13.831(3) Å. Notably, these structural distortions occur despite the nearly identical Shannon ionic radii of high‐spin Fe^3+^ and Mn^3+^ in octahedral coordination [45]. These changes can therefore be associated with Jahn–Teller distortions induced by the Mn^3+^ cation, in agreement with the quantum‐chemical calculations discussed earlier. This observation further supports lattice distortion and increased structural disorder arising from Mn incorporation into the hematite framework.

#### 2.2.6 Thermogravimetric Analysis by TGA‐DSC‐MS

TGA‐DSC‐MS measurements of an uncalcined Fe–Mn powder (${\mathrm{Fe}}_{80}{\mathrm{Mn}}_{20}{\mathrm{Ti}}_{F}$) reveal an initial mass loss of approximately 6% upon heating to 250 °C, increasing to a total mass loss of 8% at 450 °C (Figure 6). Notably, the DSC data show a pronounced exothermic peak at 199 °C (enthalpy: −104 J g^−1^). Thermal events in this temperature range are commonly attributed to the dehydroxylation of Fe and Mn hydroxide species, leading to the formation of oxide phases. The simultaneously recorded MS signals show pronounced evolution of *m*/*z* = 18 and 17, corresponding to molecular water (H_2_O) and its hydroxyl fragment (OH) generated during ionization in the mass spectrometer. Both signals exhibit a single broad release feature centered near the DSC event, indicating that the thermal transformation is dominated by one overlapping dehydroxylation process rather than multiple sequential decomposition steps. No significant evolution of additional gaseous species such as CO_2_ or O_2_ was observed within the investigated temperature range. However, dehydroxylation alone is typically reported as an endothermic process [46]. Since characteristic crystallization processes of iron oxides generally occur at significantly higher temperatures (300–500 °C) [41, 47], the exothermic peak at 200 °C is attributed to a coupled transformation involving dehydroxylation and simultaneous structural reorganization (M‐O‐M formation), nucleation, and short‐range ordering within the emerging oxide phase [48, 49]. The absence of a second crystallization peak at higher temperatures suggests that oxide formation proceeds through a single, overlapping transformation step rather than distinct sequential processes, likely resulting in a structurally disordered FeA1.5MnA0.5OA3 phase. This interpretation is consistent with the in situ HTXRD results.

**FIGURE 6:**
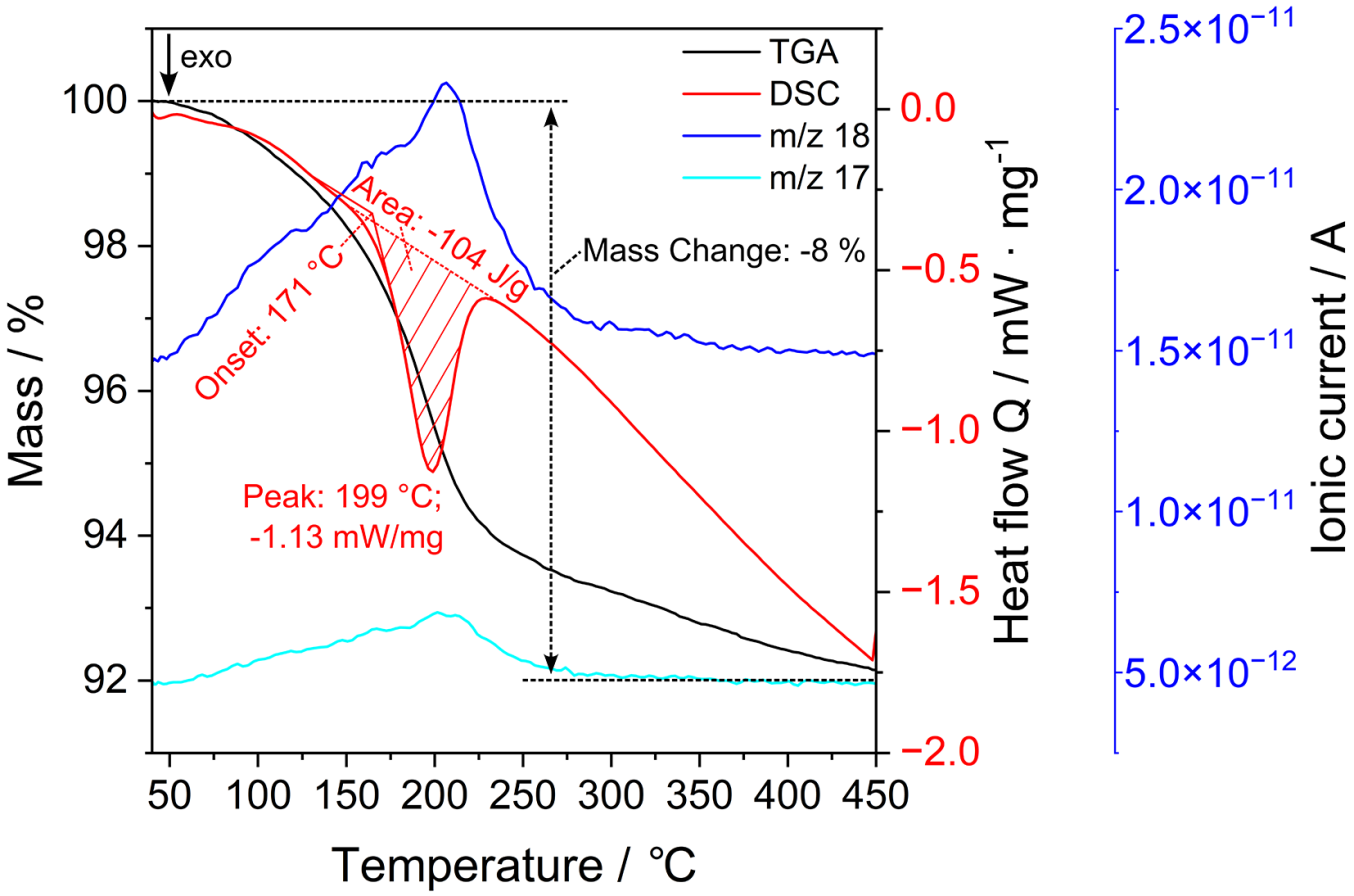
TGA‐DSC‐MS measurement of an uncalcined ${\mathrm{Fe}}_{80}{\mathrm{Mn}}_{20}{\mathrm{Ti}}_{F}$ powder sample between 40 and 450 °C at a heating rate of 10 °C min^−1^. Before the measurement, the instrument stabilized at 40 °C under a constant flow of nitrogen (20 mL min^−1^) and air (50 mL min^−1^). *m*/*z* signals of 18 and 17 correspond to the detection of molecule water (H_2_O) and hydroxyl fragment (OH), respectively. Please note additional discussion provided in the Supporting Information.

### 2.3 Post‐Test Characterization

XRD measurements were initially performed for the catalyst grown on Ti mesh (${\mathrm{Fe}}_{80}{\mathrm{Mn}}_{20}T_{350}d_{2}{\mathrm{Ti}}_{M}$) before and after electrochemical testing for 500 cycles to identify structural changes during accelerated stability testing. All measured samples show, in addition to the reflections of the Ti substrate, the presence of the hematite‐based catalyst, indicating that the material largely retains its structural characteristics after electrochemical testing (Figure S18). Additional SEM‐EDX characterization reveal similar apparent particle sizes before (44 ± 8 nm) and after electrochemical testing (46 ± 12 nm), further supporting the XRD results (Figure S17). Similarly, the Fe and Mn contents determined by EDX remain within comparable ranges before and after testing, with Fe ranging from 72 to 77 at.% and Mn from 23 to 28 at.%. No systematic change in the Fe:Mn ratio is observed, indicating no pronounced change in the local catalyst composition during the 500‐cycle stability test.

To further evaluate the effect of extended stability testing under more relevant operating conditions, the same physicochemical characterizations were performed on the electrode tested at 50 mA cm^−2^ for 77 h. XRD measurements of the tested electrode show the same characteristic reflections of the hematite‐based catalyst observed for the fresh electrode (Figure S17), confirming that the crystalline hematite‐based FeA1.5MnA0.5OA3 phase remains detectable after extended electrochemical operation. However, distinct differences between the fresh and aged electrodes cannot be reliably resolved by laboratory XRD owing to the low catalyst loading and thin catalyst layer on the Ti mesh support.

In contrast, SEM analysis reveals substantial changes in the apparent particle size after extended electrochemical operation, increasing to 230 ± 60 nm (Figure S17), compared with approximately 45 nm for the fresh electrode. The semi‐quantitative SEM‐EDX analysis further shows an Fe:Mn ratio close to 1:1 after testing (Figure S17), compared with the initial ratio of approximately 3:1. ICP‐OES analysis of the electrolyte detect 0.75 ppm Fe and 0.10 ppm Mn, indicating substantially higher Fe dissolution than Mn dissolution. The preferential dissolution of Fe therefore provides a plausible explanation for the observed shift in the remaining catalyst composition toward lower Fe contents and is consistent with the degradation behavior previously reported for this catalyst system [13].

### 2.4 Mechanistic Insights Into the Oxygen Evolution Reaction From Operando Raman

To elucidate the mechanism of OER, operando Raman spectroscopy was used, a widely utilized method to investigate the structural stability and surface dynamics of electrocatalysts under operating conditions [50]. The electrochemical data can be found in Figure S18. At OCP, the spectra exhibit the characteristic hematite bands (223, 291, 406, 498, 610, 660, and 1321 cm^−1^), consistent with the corresponding ex situ measurements, although with reduced intensity due to the presence of electrolyte on the electrode surface (Figure 7). Additional features at 933 and 1642 cm^−1^ are attributed to symmetric stretching of ClOA4A− ions and the bending mode of molecular water, respectively [51, 52].

**FIGURE 7:**
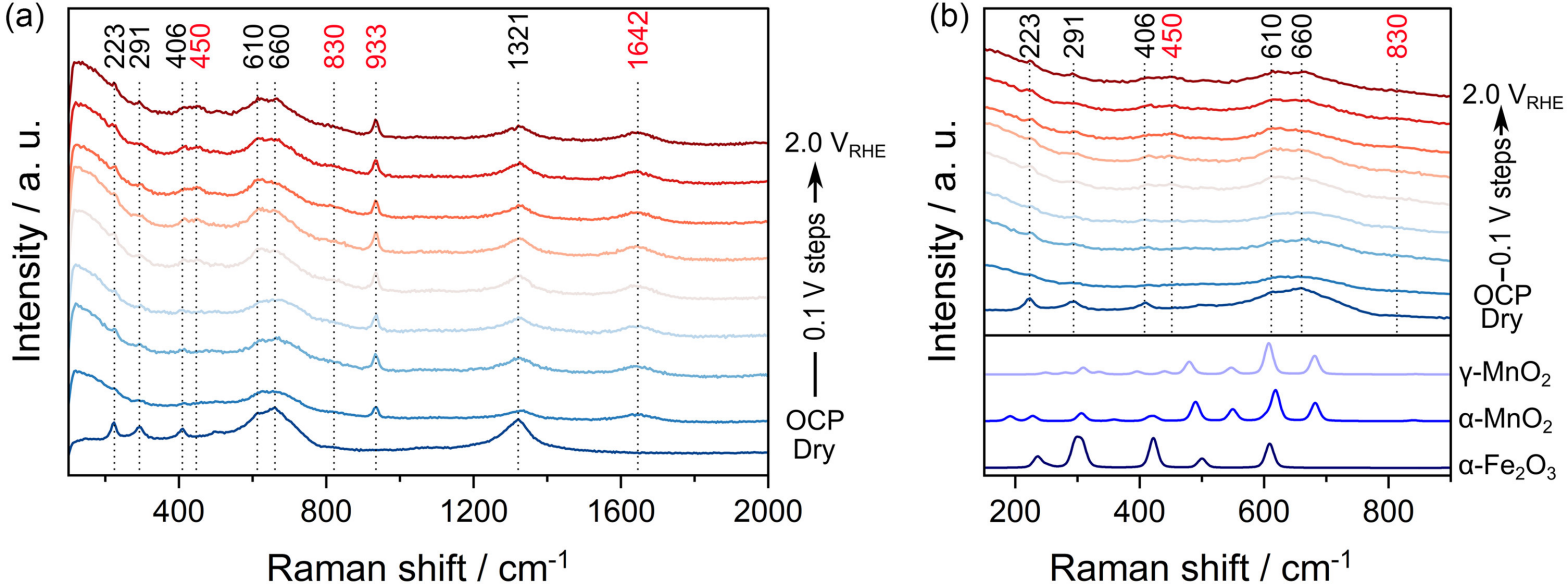
Operando Raman spectra of an ${\mathrm{Fe}}_{80}{\mathrm{Mn}}_{20}T_{350}d_{2}{\mathrm{Ti}}_{F}$ electrode recorded during potential holds between OCP and 2.0 V vs. RHE in 0.1 M HClOA4 at room temperature to monitor potential‐dependent structural changes of the Mn‐substituted hematite phase under OER conditions. Measurements were performed using a Ag/AgCl (3 M KCl) reference electrode and a Pt wire counter electrode. At each potential, three spectra were accumulated in the range of 100–2000 cm^−1^ using a 532 nm excitation wavelength at 50% laser power. (a) Full spectra and (b) enlarged region between 150 and 900 cm^−1^, including DFT‐calculated Raman spectra of *α*‐Fe_2_O_3_ and *α*‐ and *γ*‐MnO_2_.

Upon increasing the potential to 2.0 V_RHE_, the positions of the hematite‐derived bands remain essentially unchanged, indicating the structural persistence of the Fe–Mn oxide host under acidic OER conditions. This observation is consistent with previous operando studies on iron oxides, where bulk lattice integrity is maintained while catalytic activity originates from surface‐localized transformations [53]. However, normalization of the spectra reveals clear potential‐dependent intensity variations near the OER onset. In particular, between 1.6 and 1.9 V_RHE_, a new feature emerges in the 430–470 cm^−1^ region that is absent under ex situ conditions (Figure 7). Bands in this range are commonly associated with metal–oxygen vibrations in distorted MOA6 octahedra and with the formation of MOOH‐like surface phases [54].

In iron oxides, similar signatures have been linked to the formation of FeOOH‐like environments under anodic polarization [55]. In manganese‐containing systems, this spectral region has been correlated with oxidation of Mn^3+^ to Mn^4+^ and structural rearrangements toward MnOOH‐ or birnessite‐type motifs under OER‐relevant potentials [54, 56]. Notably, reported Mn^3+^/Mn^4+^ redox transitions occur in a similar potential window (1.6–1.7 V_RHE_), coinciding with the OER onset observed here.

These observations suggest the formation of high‐valent surface metal‐oxo species during OER operation. However, Raman spectroscopy alone does not allow an unambiguous assignment of either Fe‐ or Mn‐centered active sites. Two mechanistic scenarios discussed in the literature may rationalize the present findings. Several operando studies identify the Mn^3+^/Mn^4+^ redox flexibility in disordered manganese oxides as a key contributor to OER activity [54, 57]. The structural adaptability of Mn‐based oxyhydroxide motifs enables dynamic lattice oxygen participation and rapid redox cycling. The emergence of the 430–470 cm^−1^ feature within the Mn redox potential window therefore supports the possibility that Mn centers directly participate in catalytic turnover. Alternatively, studies on Fe–Mn spinel systems such as FeMnA2OA4 propose Fe^3+^ as the primary active center, while Mn^3+^ promotes water adsorption and oxygen donation through electronic interaction with Fe sites [58]. Although these studies were conducted in alkaline media and involve different formal oxidation states, a similar cooperative interaction between Fe and Mn cannot be excluded here. Within this interpretation, Mn incorporation modifies the Fe–O electronic structure and covalency, thereby stabilizing high‐valent Fe‐oxo intermediates during OER operation. This interpretation is consistent with the increasing intensity of the hematite‐derived *E*_g_ mode at approximately 610 cm^−1^ with rising potential (Figure 7). This vibrational mode corresponds to symmetric Fe–O stretching within the FeOA6 framework and is sensitive to changes in Fe–O covalency and local coordination [59, 60]. The concurrent evolution of the 430–470 and 610 cm^−1^ bands therefore most likely reflects the formation of a thin catalytically active FeOOH surface layer, while the hematite bulk lattice remains largely preserved.

A band at 830 cm^−1^ is also observed (Figure 7). This feature has been assigned to O─O or OOH vibrations [61], surface peroxo/superoxo intermediates [62], or, in Mn systems, to MnOA4A− species [63]. Considering that Mn dissolution studies under comparable acidic conditions indicate suppressed MnOA4A− formation [13], the accumulation of oxygen‐derived intermediates appears more consistent with the present data. The weak feature at 1050 cm^−1^ further supports the formation of oxygen‐derived species such as OA2A2− or related intermediates during OER [52].

Overall, the operando Raman results indicate structural persistence of the hematite‐based host lattice accompanied by the formation of high‐valent surface oxyhydroxide species under anodic bias. The coincidence of OER onset with the possible Mn^3+^/Mn^4+^ transition may suggest that Mn promotes water adsorption and surface intermediate formation while simultaneously electronically modifying neighboring Fe sites. At the same time, the pronounced response of Fe–O vibrational modes suggests the direct electronic involvement of Fe centers during catalytic turnover [64, 65]. However, additional operando measurements such as XPS or XAS in combination with quantum‐chemical calculations on the OER mechanisms are needed to fully interpret the OER mechanism for FeA1.5MnA0.5OA3 in acidic media.

## 3 Conclusion

A structurally disordered FeA1.5MnA0.5OA3 catalyst forms during calcination through the transformation of poorly crystalline iron oxide/hydroxide and MnOA2 precursor species. Raman spectroscopy reveals an increasing intensity and broadening of the symmetry‐forbidden mode in hematite at 660 cm^−1^ after calcination, indicating the formation of substitution‐induced structural disorder. This observation is supported by XRD and TEM measurements, which show clear differences in diffraction features between FeA2OA3, calcined FeA1.5MnA0.5OA3, and the corresponding uncalcined precursor material. Quantum chemical calculations and synchrotron WAXS further confirm that Mn incorporation induces lattice distortions through Jahn–Teller effects and variations in Fe─O and Mn─O bond lengths.

The structural characterization results correlate with the observed electrochemical behavior. Calcination between 250 and 350 °C leads to complete dehydroxylation and formation of a disorder‐rich Mn‐substituted hematite phase. This appears to enhance the adsorption behavior of oxygen evolution intermediates and thereby improve catalytic activity, with an optimum calcination condition identified at 350 °C for 2 h. In contrast, higher calcination temperatures (≥450 °C) increase crystallinity and structural ordering, as confirmed by in situ HTXRD and the sharpening of the Raman band at 660 cm^−1^, resulting in reduced catalytic activity due to less favorable adsorption properties. These results suggest that calcination temperature affects catalytic performance of FeA1.5MnA0.5OA3 by tuning the adsorption characteristics of the catalyst surface through substitution‐induced lattice disorder.

Operando Raman measurements suggests that both Fe and Mn participate in the catalytic process. Future operando XPS and XAS investigations will further clarify the electronic structure evolution and reaction mechanism during operation.

## 4 Experimental Section

### 4.1 Catalysts Synthesis

Ti mesh (Hongyunda Metal Products Limited Company) was cut into 2 cm × 2 cm pieces and boiled in concentrated hydrochloric acid (32 wt.‐%, Sigma–Aldrich) for 10 min. The mesh was removed from the acid and washed several times with Milli‐Q deionized water (18.2 MΩ cm at 25 °C) and ethanol (≥94.0%, Etax A) before being left to dry. The catalysts were grown on the Ti mesh using a hydrothermal approach. A total of 10 mmol of stoichiometric amounts of Fe(NOA3)A2⋅9HA2O (≥98%, Sigma–Aldrich) and KMnOA4 (98%, Thermo Fisher) were dissolved in 30 mL of deionized water while adding 0.01 mmol of citric acid monohydrate (≥99.5%, Sigma–Aldrich). The stoichiometry used for the optimization of catalyst calcination temperature was a molar ratio of 80:20 of Fe:Mn, while the molar ratio was changed to 100:0 for the synthesis of pure hematite (Table S5). The solutions were stirred for about 30 min to ensure good mixing and transferred to a 50 mL Teflon‐lined stainless steel autoclave, in which the Ti mesh was inserted. The reaction vessel was sealed and heated in an oven at 140 °C for 12 h. Afterward, the hydrothermal vessel was left to cool to room temperature, and the electrode was removed and washed several times with water and ethanol.

The electrodes were dried in an oven overnight at 80 °C. The air calcination was carried out at temperatures between 209 and 491 °C for durations between 0.6 and 3.4 h (Table S5). The electrodes were cut in half (1 cm × 2 cm) for simultaneous physicochemical and electrochemical investigations.

The average mass increase after hydrothermal synthesis was determined from five independently prepared electrodes and amounted to 1.8 ± 0.16 mg per electrode. This average coating mass was used to estimate the catalyst mass loading for the calculation of mass activities.

For Raman and operando Raman spectroscopy, the catalyst was grown on Ti foil (Hongyunda Metal Products Limited Company) using the same procedure described before and using 4 mmol of metal salts (Table S5).

For thermogravimetric analyses, the synthesis was repeated in the same manner without the addition of a Ti mesh. The powder obtained was washed with water and ethanol in a centrifuge at 8000 RPM for 10 min and then dried at 80 °C overnight.

### 4.2 Catalyst Characterization

Raman spectroscopy was performed using a Renishaw inVia Qontor with a laser wavelength of 532 nm and a power of 5%. The catalysts supported on a Ti mesh and foil were measured between 100 and 2000 cm^−1^ at three different spots with three accumulations each.

Transmission electron microscopy (TEM) measurements were performed using a JEOL JEM‐2800 microscope with an accelerating voltage of 200 kV. The on Ti foil grown materials were mechanically removed, dispersed in ethanol, and drop‐casted on copper grids (AGS147‐3, Agar Scientific).

All catalyst morphology and relative quantification were analyzed by a Zeiss Sigma VP scanning electron microscopy (SEM) instrument with a Schottky FEG emitter as the electron source. The catalysts supported on Ti mesh or foil were mounted on a sample holder with carbon tape. The SEM images and semiquantitative compositions by EDX were measured at an accelerating voltage of 8 kV. Cross‐sectional images were performed using the same instrument. Ti mesh electrodes were polished and clamped into a cross‐sectional sample holder. Particle diameters were manually determined from SEM images using ImageJ by measuring the apparent diameter of individual particles.

Inductively coupled plasma optical emission spectrometer (ICP‐OES) analysis was performed to investigate the material composition. Therefore, about 50 mg of powder material was digested in 5 mL of deionized water, 2 mL of HCl (34%–37%, ROMIL), and 6 mL of HNO_3_ (67%–69%, ROMIL) and transferred into a 100 mL microwave (Berghof Speedwave Xpert) vessel. A blank sample with only the acid solution was prepared. The vessel was closed and heated to 200 °C for 30 min, after heating to 225 °C for 40 min, and finally at 250 °C for 40 min. The solutions were filled to 50 mL with deionized water and further diluted to obtain final Fe and Mn concentrations between 1 and 4 ppm. Multistandard solutions (Labking, Multi‐element solution 33 components, 100 ppm) were used to prepare standards with concentrations of 0.2, 1, 3, 6, and 10 ppm. An Agilent Inc 5900 SVDV ICP‐OES was used in axial and radial reading mode by a single detector (CCD array 167–785 nm) for the elemental analysis.

XRD measurements were performed using an X’pert Pro MPD (*λ* = 1.5406, CuKα; 45 kV, 40 mA). The electrodes were measured at 2*θ* = 10°–80°. In situ HTXRD measurements for the catalyst grown on Ti foil were conducted with the same instrument, combined with an oven (HTK 1200N, Anton Paar, Australia). The electrodes were heated from room temperature to 450 °C and then cooled to room temperature for the catalyst grown on a Ti foil. Every 50 °C, an XRD pattern was acquired between 2*θ* = 10°–80°. The same procedure was repeated for a pure Ti foil after hydrothermal treatment without the addition of metals. Measurements were also performed for a catalyst grown in a Ti mesh up to a temperature of 550 °C and for a pure Ti mesh.

Synchrotron wide‐angle X‐ray scattering (WAXS) measurements were performed for pure iron oxide and iron–manganese mixed oxide samples calcined at 350 °C for 2 h. The electrodes were cut into smaller pieces, mounted in sample compartments, and sealed with 50 μm Kapton foil. Due to the high photon flux of the synchrotron source (*λ* = 0.165 Å), short exposure times of approximately 1 s per sample were sufficient. The resulting 2D diffraction images were azimuthally integrated and stored as 1D XRD patterns in HDF5 format for subsequent analysis. Le Bail‐type profile fitting of the obtained diffraction patterns were performed using the FullProf software suite to determine lattice parameters and structural changes using *α*‐Fe_2_O_3_ ($R\bar{3}c$) and *α*‐Ti (*P*6_3_/mmc) as reference crystal structures.

Thermal analysis was done for a powder sample using a Netzsch STA 449 F3 Jupiter TG‐DSC (coupled with a QMS 403Aëolos Quadro) under constant air flow (50 mL min^−1^). The sample in an alumina crucible was stabilized at 40 °C, heated to 450 °C at a rate 10 °C min^−1^, and kept at the target temperature for 30 min before cooling back to 40 °C.

### 4.3 Quantum Chemical Calculations

Quantum chemical calculations were performed for FeA2OA3, FeA1.5MnA0.5OA3, and Mn oxides with the PBE0 hybrid density functional method (DFT‐PBE0) [66, 67] using the CRYSTAL23 program package [68]. Gaussian‐type triple‐*ζ*‐valence + polarization (TZVP) level basis sets were applied for all atoms. The basis sets for Fe, Mn, and O have been previously derived from the molecular Karlsruhe def2 basis sets [69, 70]. For the evaluation of the Coulomb and exchange integrals (TOLINTEG), tight tolerance factors of 8, 8, 8, 8, and 16 were used. Both the atomic positions and lattice constants were fully optimized under the constraints of space‐group symmetry, using the default optimization convergence criteria in CRYSTAL.

The structural optimization and magnetic ground state evaluation of *α*‐Fe_2_O_3_, the investigated MnOA2 polymorphs, MnA2OA3, and MnA3OA4 have previously been carried out in Ref. [70]. Ordered models of Mn‐substituted *α*‐Fe_2_O_3_ ($R\bar{3}c$), named a‐Fe_1.5_Mn_0.5_O_3_ and b‐Fe_1.5_Mn_0.5_O_3_, were constructed based on a supercell of *α*‐Fe_2_O_3_. *α*‐Fe_2_O_3_ has 10 atoms in the primitive cell. To accommodate Mn substitution and magnetic ordering, the primitive cell had to be increased along one of the equal primitive lattice vectors. This results in a triclinic crystal structure (*P*1) with 20 atoms in the cell for a‐Fe_1.5_Mn_0.5_O_3_ and b‐Fe_1.5_Mn_0.5_O_3_. The FeA1.5MnA0.5OA3 composition was chosen due to its proximity to the estimated sample composition. Furthermore, computational limitations are imposed by the increased supercell size needed for more complex Mn substitutions. The magnetic ground state of both ordered models was found to be ferrimagnetic, with a ferromagnetic ordering for the Mn atoms and an antiferromagnetic ordering for the Fe atoms. Further order types were explored, but the SCF calculations did not converge in these cases. Optimized crystal structures in the CIF format, magnetic moments calculated for Fe and Mn atoms (Table S6), and the Monkhorst–Pack‐type *k*‐meshes [71] used for sampling of the reciprocal space are given in the Supporting Information.

The harmonic vibrational frequencies and Raman intensities were obtained by using the computational schemes implemented in CRYSTAL [72, 73, 74, 75]. A small imaginary vibrational mode of 66*i *cm^−1^ persisted for b‐Fe_1.5_Mn_0.5_O_3_. Attempts to distort the structure along this mode resulted in even larger imaginary frequencies. Imaginary frequencies were not observed in the other structures studied. The Raman intensities were calculated for a polycrystalline powder sample (total isotropic intensity in arbitrary units). The Raman spectra were obtained by using a pseudo‐Voigt band profile (50:50 Lorentzian:Gaussian) and an FWHM of 16 cm^−1^. The experimental laser wavelength of 532 nm and temperature of 298.15 K were used.

### 4.4 Electrochemical Characterization

#### 4.4.1 Measurements Using a Three‐Electrode Setup

All electrochemical measurements for the DOE optimization were performed in a standard three‐electron setup using a curled platinum wire as the counter electrode and a reversible hydrogen electrode (RHE, Gaskatel) as the reference electrode in 0.1 M HClOA4, diluted from concentrated HClOA4 (60 wt.‐%, Sigma–Aldrich, p.a). The electrolyte temperature was controlled by an outer water bath, set to 25 °C, and measurements were performed with a potentiostat/galvanostat (Autolab PGSTAT12). The electrolyte was saturated before each measurement by bubbling N_2_ for 30 min and N_2_ surface flow during the measurement. The electrodes were immersed about 0.5 cm in the electrolyte, achieving an approximate total geometric area of 0.5 cm^2^ (0.5 × 1 cm).

To evaluate the initial activity of the catalysts toward the OER, three cyclic voltammetry (CV) measurements were performed between 1.4 and 2.0 V_RHE_. In addition, impedance spectroscopy (EIS) was recorded for materials between 10 kHz and 0.1 Hz, with an AC amplitude of 10 mV at 1.5, 1.7, and 1.9 V_RHE_. The catalyst stability was investigated by an accelerated stress test (AST). Therefore, the catalyst was cycled 500 times between 1.4 and 2.0 V_RHE_ with a scan rate of 100 mV  s^−1^. The measurement was interrupted every 100 cycles to perform a slow scan CV (10 mV  s^−1^) between 1.4 and 2.0 V_RHE_ and an EIS measurement at 1.9 V_RHE_ in the same manner as previously described. For all relevant samples, EIS measurements were conducted at open circuit potential (OCP) to determine double‐layer capacitances (*C*_dl_) and electrochemically active surface area (ECSA) by comparing with a flat surface (0.02 mF cm^−2^ [21, 76]). For each synthesis condition, two independently prepared electrodes were measured to verify the reproducibility of the electrochemical performance. The reported error bars represent the standard deviation of these independent measurements.

An equivalent electrical circuit was used to fit the EIS data, which can be found in the Figure S2, and can be formulated as follow, using the Boukamp notation: *R*_Ω_ (*R*_f_ *C*_f_) (*C*_dl_ *R*_ct_ (*C*_a_ *R*_a_)) [77]. The *C*_dl_ was substituted by a constant phase element (CPE), originating from the surface roughness and porosity, where *Z*_CPE_ is given by

(1) $$Z_{\mathrm{CPE}}=\frac{1}{Q{\left(j\omega \right)}^{n}}$$

*Q* and *n* are parameters of the CPE, while *j* is the imaginary number, and *ω* the angular frequency. The actual double‐layer capacitance was calculated from the CPE parameters by a method proposed by Hsu and Mansfeld [78]:

(2) $$C=Q{\left(\omega_{m}^{\prime \prime }\right)}^{n-1}$$

$\omega_{m}^{\prime \prime }$ describes the angular frequency when the imaginary part of the impedance is maximum. The uncompensated ohmic resistance (*R*_Ω_) was determined by equivalent circuit fitting of the EIS data. Unless stated otherwise, all electrochemical potentials reported in this work are iR‐corrected using the corresponding calculated iR drop.

Additional long‐term stability measurements were performed at 50 mA cm^−2^ for 77 h using the same three‐electrode setup described above. The measurement was interrupted every 10 h to record a polarization curve between 0 and 50 mA cm^−2^ and an EIS spectrum at 50 mA cm^−2^. The electrolyte level was marked at the beginning of the measurement and restored daily using the same electrolyte to compensate for electrolyte consumption and evaporation and thereby maintain a constant electrode immersion area. At the end of the stability measurement, the electrolyte was collected for ICP‐OES analysis to quantify dissolved Fe and Mn species.

#### 4.4.2 Operando Raman Measurements

Operando Raman measurements were performed in a Raman electrochemical flow cell (redox.me, 3.5 cm^2^, volume 4.5 mL). The cell is equipped with a Pt wire counter and a Ag/AgCl 3 M KCl electrode (Metrohm). A catalyst grown on Ti foil (${\mathrm{Fe}}_{80}{\mathrm{Mn}}_{20}T_{350}d_{2}{\mathrm{Ti}}_{F}$) was placed in the cell. The cell was closed, and 0.1 M HClOA4, prepared as described above, was pumped into the slightly tilted cell to remove all gas bubbles until the cell was filled. The cell was placed under the Raman spectrometer measurement laser (Renishaw inVia Qontor). To verify electrochemical connection and electrode performance, an EIS was measured between 10 kHz and 1 Hz, followed by a CV between 1.2 and 2.0 V_RHE_ with a scan rate of 10 mV  s^−1^. All operando Raman measurements were done with a laser wavelength of 532 nm and a power of 50% between 100 and 2000 cm^−1^. Each measurement consisted of three accumulations on the same area. After the initial Raman measurement at the OCP, the cell potential was stepped from 1.20 to 2.10 V (1.40 to 1.96 V_RHE_) in 100‐mV increments, with three Raman spectrum accumulations acquired for each step.

## Author Contributions

**Moritz Karl Rosenthal** **:** conceptualization, investigation, formal analysis, methodology, Visualization, funding acquisition, writing – original draft preparation. **Kim Eklund** **:** investigation, formal analysis, methodology, writing – original draft preparation. **Eeva‐Leena Rautama** **:** conceptualization, formal analysis, supervision, writing – review and editing. **Lilian Moumaneix** **:** conceptualization, methodology, formal analysis, supervision, writing – review and editing. **Polina Kalachikova** **:** methodology, investigation, review and editing. **Nastaran Farrahi** **:** investigation, formal analysis, writing – review and editing. **Xiangze Kong** **:** methodology, investigation, review and editing. **Antti J. Karttunen** **:** methodology, writing – review and editing. **Tanja Kallio** **:** conceptualization, methodology, project administration, supervision, funding acquisition, writing – review and editing.

## Funding

This work was funded by the Neste and Fortum foundation (Grant Number 20250002), the Research Council of Finland (Project Number 352955), and the European Synchrotron Radiation Facility (Grant IHCH1890).

## Conflicts of Interest

The authors declare that they have no known competing financial interests or personal relationships that could have appeared to influence the work reported in this paper. The findings described in this manuscript are part of a pending PCT patent application (Application No. PCT/FI2026/050227) filed by Aalto University.

## Supporting information

Supplementary Material

## Data Availability

Data will be made available upon request.
