# Supplementary material for: Insights Into the Oxygen Evolution Reaction on an Mn‐Bearing Hematite Electrocatalyst in an Acidic Electrolyte
Source: ChemSusChem. 2026 Oct 4;19(19):e71101. doi: 10.1002/cssc.71101 (PMC13635278; doi:10.1002/cssc.71101)
Supplement: Supplementary file 1 [file CSSC-19-e71101-s001.pdf]

Supporting Information

Insights into the Oxygen Evolution Reaction on an Mn-Bearing Hematite Electrocatalyst in an Acidic Electrolyte

Moritz Karl Rosenthal[a] | Kim Eklund[a] | Eeva-Leena Rautama[a] | Lilian Moumaneix[a] | Polina Kalachikova[a] | Nastaran Farrahi[a] | Xiangze Kong[a] | Antti J. Karttunen[a] | Tanja Kallio[a]

[a]*Department of Chemistry and Material Science, School of Chemical Engineering, Aalto University, Kemistintie 1, Espoo, 02150, Finland*

Results and Discussion

Electrochemical Characterization

Electrochemical mass activity

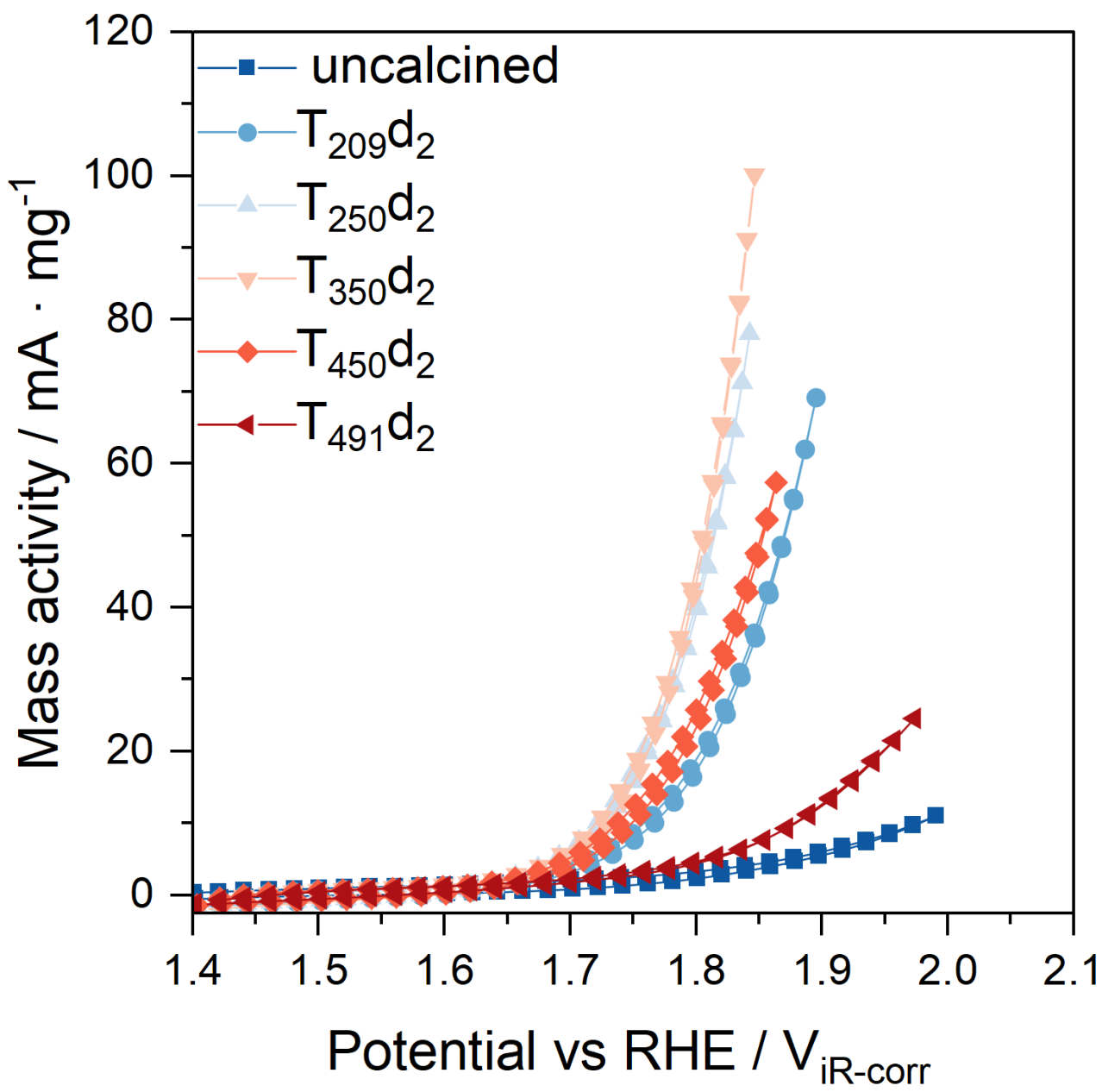

Figure S1: OER mass activity of the Fe–Mn catalysts grown on Ti mesh, measured in a three-electrode cell using a reversible hydrogen electrode (RHE) as the reference electrode and a Pt wire as the counter electrode in 0.1 M $HClO_4$ at 25 °C. The average catalyst coating mass after hydrothermal synthesis was determined to be 1.8 ± 0.16 mg for a 2 × 2 cm Ti substrate (4 cm^2). Assuming homogeneous catalyst deposition, the catalyst loading within the electrochemically exposed area (0.50 cm^2) was estimated to be 0.225 ± 0.020 mg, which was used to calculate mass activity.

Electrochemical impedance spectroscopy (EIS) measurements

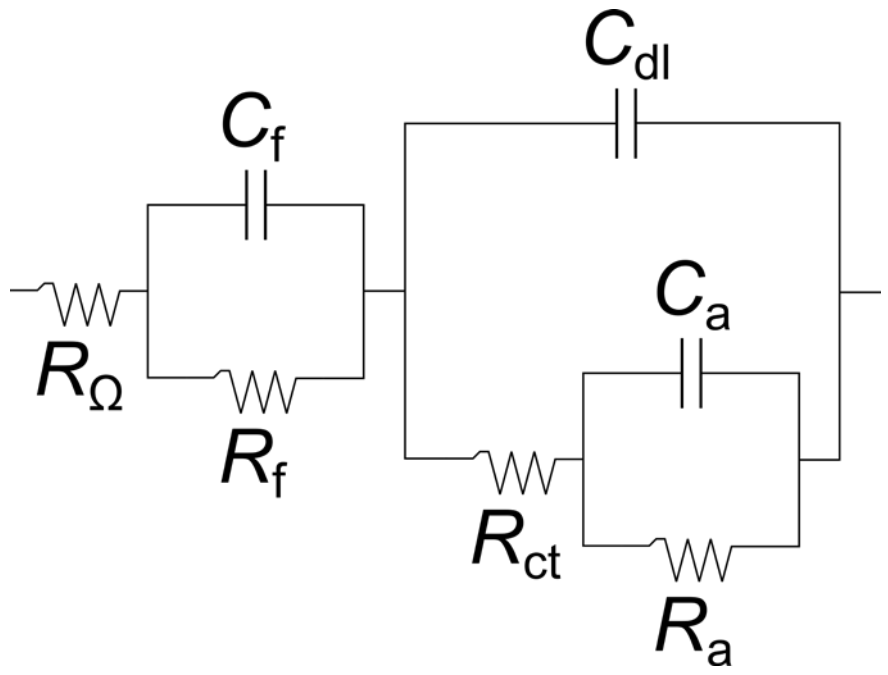

Figure S2: Equivalent circuit model used for fitting the EIS data of the investigated electrodes. The circuit consists of the ohmic resistance of the electrolyte and cell setup R_Ω in series with a parallel film resistance–capacitance element $R_f \parallel C_f$ representing the Ti mesh substrate, followed by a parallel combination of the double layer capacitance C_{dl}, replaced in the model with a constant phase element (CPE), and the charge-transfer resistance R_{ct}. In addition, a parallel adsorption resistance–capacitance element, also replaced with a CPE, $R_a \parallel C_a$ was included to account for surface adsorption processes.

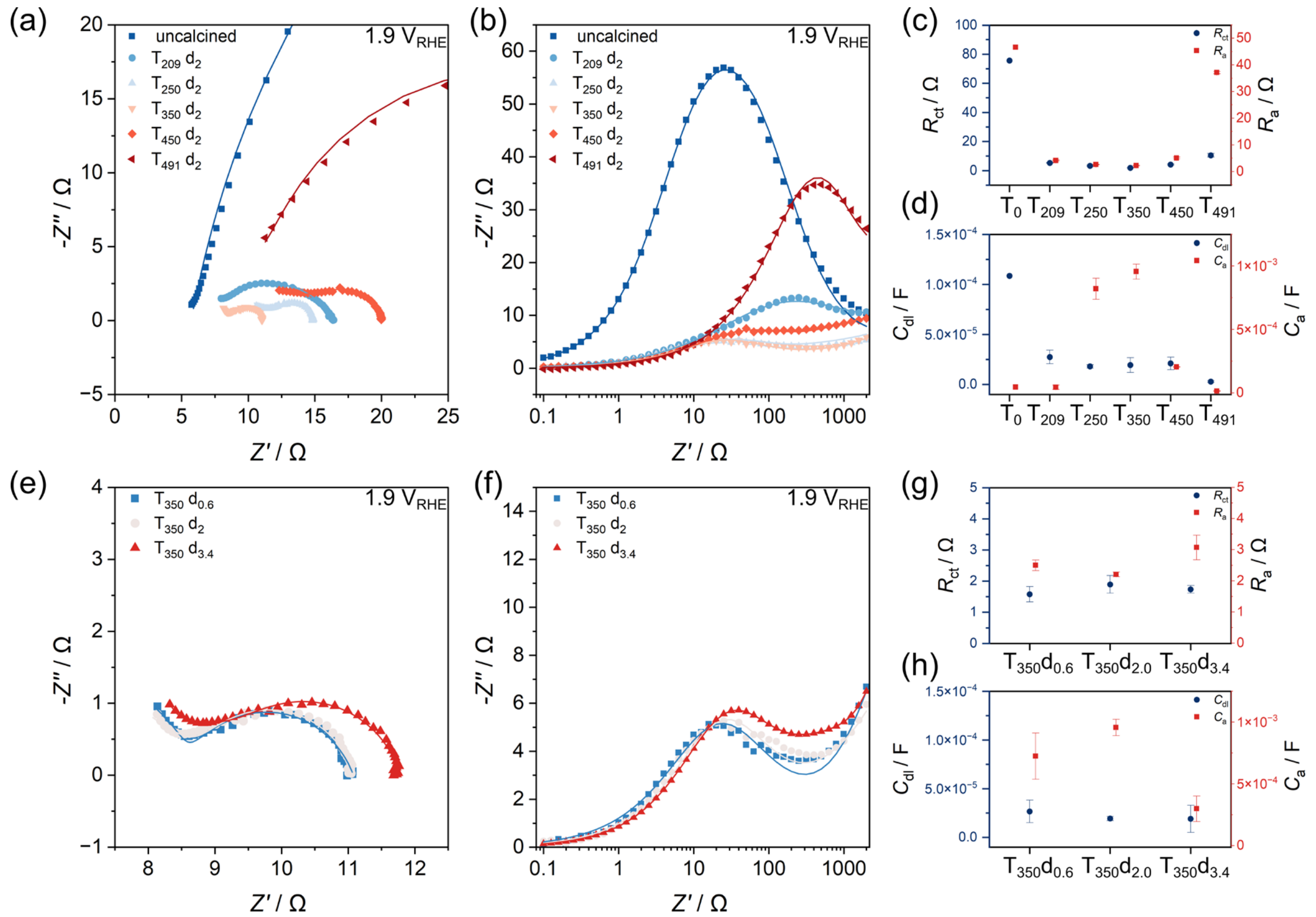

Figure S3: Electrochemical impedance spectroscopy (EIS) measurements for the catalysts grown on the Ti mesh recorded in a three-electrode configuration using a reversible hydrogen electrode (RHE) as a reference electrode and a Pt wire as a counter electrode in 0.1 M $HClO_4$ at 25 °C. The influence of calcination temperature on the impedance response measured at 1.9 V_{RHE} (uncompensated) is shown as (a) Nyquist plots (symbols) with corresponding equivalent-circuit fits (solid lines) and (b) Bode plots (symbols) with corresponding equivalent-circuit fits (solid lines). The fitted parameters R_{ct}, R_a, C_{dl}, and C_a are summarized in (c) and (d). The influence of calcination duration on the impedance response is shown as (e) Nyquist plots (symbols) with corresponding fits (solid lines) and (f) Bode plots (symbols) with corresponding fits (solid lines). The fitted parameters R_{ct}, R_a, C_{dl}, and C_a are summarized in (g) and (h).

Electrochemical active surface area (ECSA)

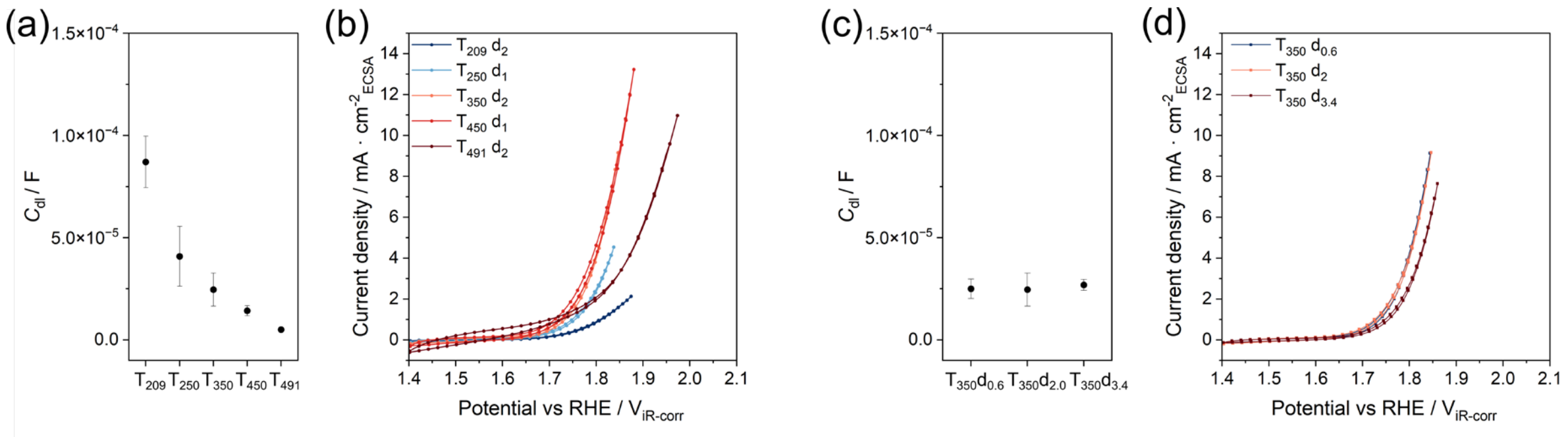

Figure S4: Effective double-layer capacitance values derived from the constant phase element (CPE) fitting of the electrochemical impedance spectroscopy (EIS) data recorded at open-circuit potential (OCP) for the catalysts grown on the Ti mesh in a three-electrode configuration using a RHE as a reference electrode and a Pt wire as a counter electrode in 0.1 M $HClO_4$ at 25 °C. The capacitance values were used as an estimate of the ECSA by comparison with the specific capacitance of a planar oxide surface (0.02 mF·cm^{-2} [1, 2]). (a) Effective double-layer capacitance values of the samples calcined at different temperatures and (b) the corresponding ECSA-normalized cyclic voltammograms (CVs) measured with scan rates of 10 mV · s^{-1}. (c) Effective double-layer capacitance values of the samples calcined for different durations and (d) the corresponding ECSA-normalized CVs measured with scan rates of 10 mV · s^{-1}.

Electrochemical stability

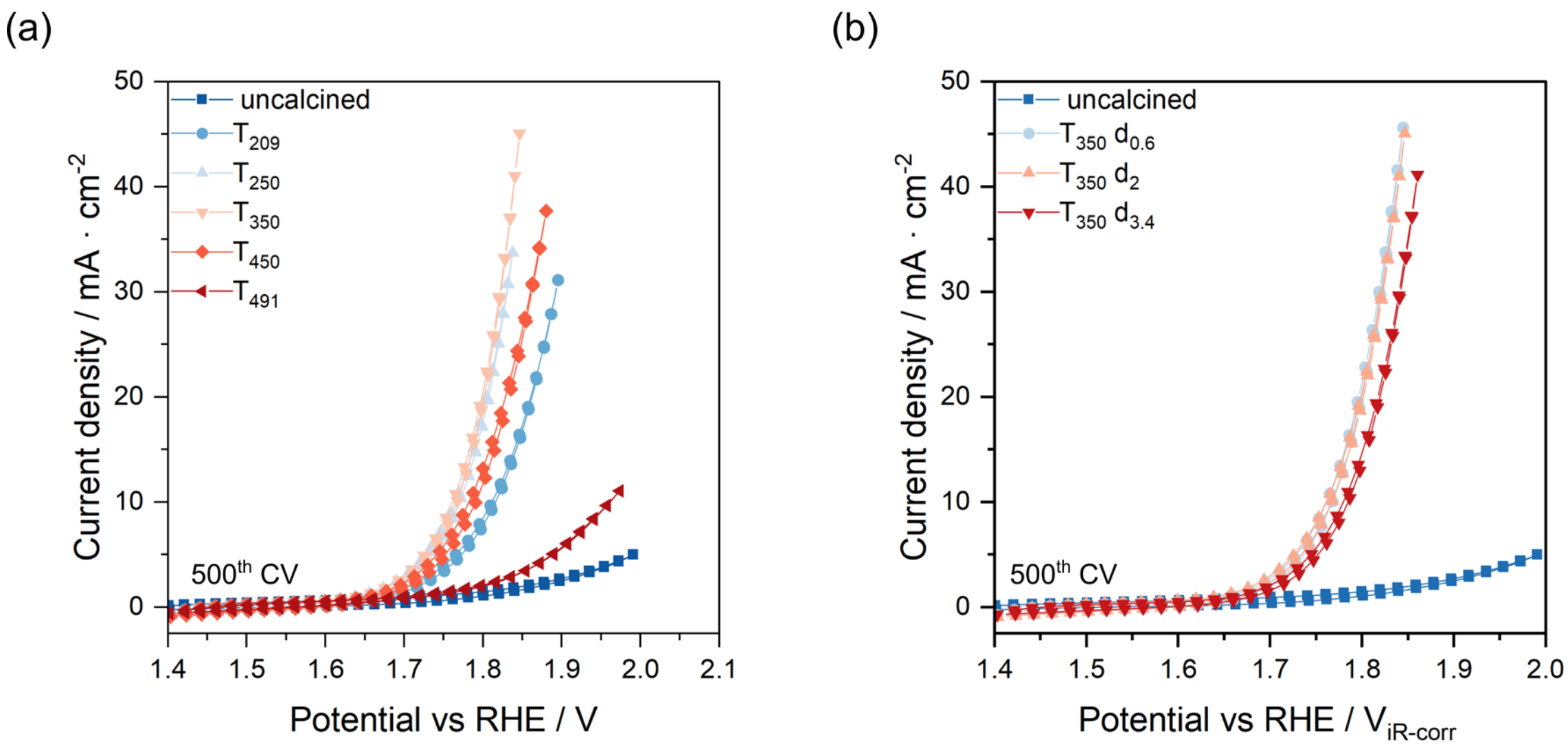

Figure S5: Electrochemical performance of the catalysts grown on the Ti mesh after 500 CV cycles recorded between 1.4 V_{RHE} and 2.0 V_{RHE} at a scan rate of 100 mV · s^{-1} in a three-electrode configuration using a RHE as a reference electrode and a Pt wire as a counter electrode in 0.1 M $HClO_4$ at 25 °C. (a) Cyclic voltammograms recorded between 1.4 V_{RHE} and 2.0 V_{RHE} at a scan rate of 10 mV · s^{-1} for the catalysts calcined at different temperatures after stability testing, and (b) CVs recorded under identical conditions for the catalysts calcined for different durations.

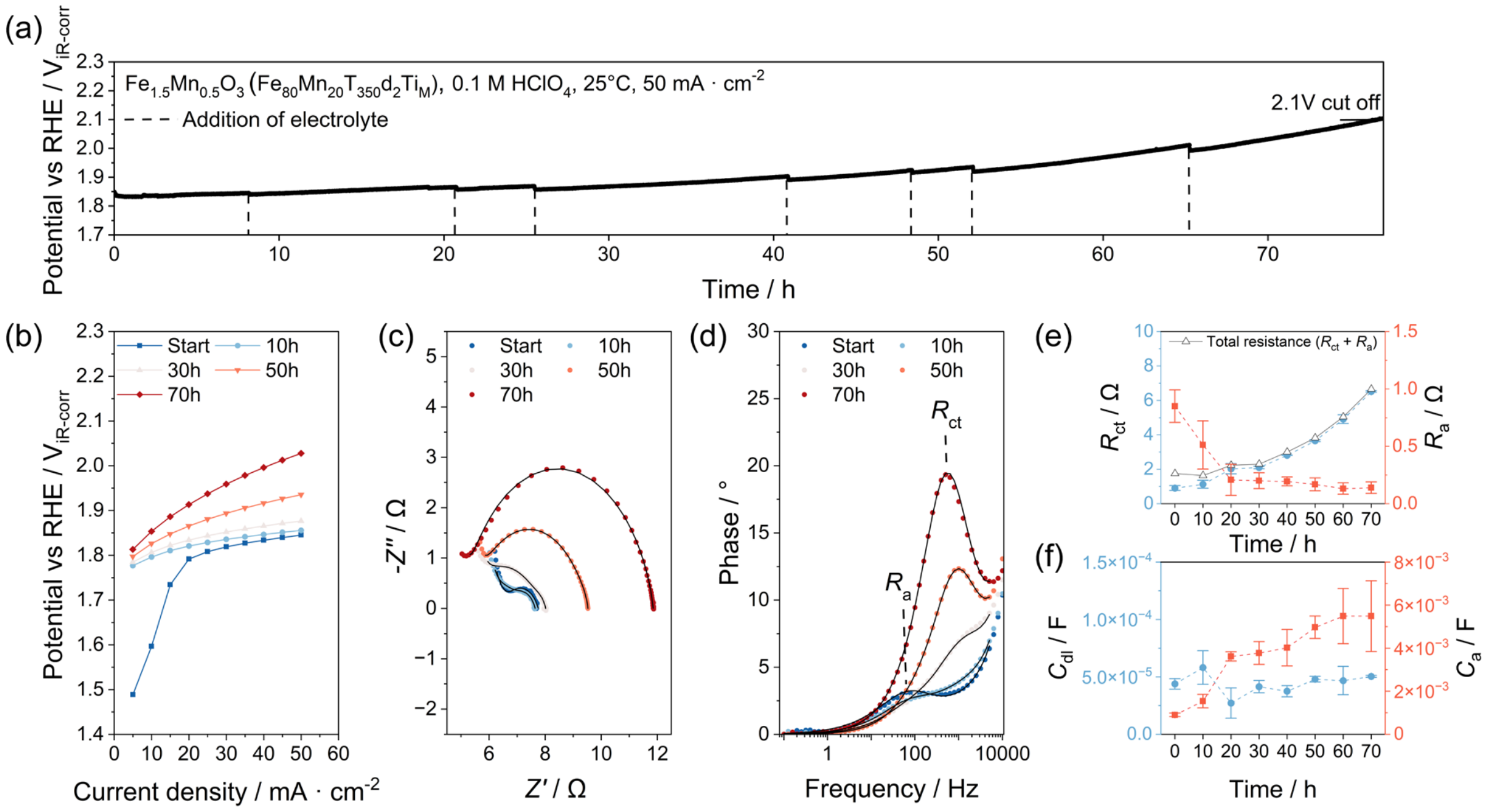

Figure S6: Extended stability test of the best-performing catalyst grown on Ti mesh ($Fe_{80}Mn_{20}T_{350}d_2Ti_M$) at 50 mA · cm^{-2} for 77 hours in a three-electrode configuration using a RHE as a reference electrode and a Pt wire as a counter electrode in 0.1 M $HClO_4$ at 25 °C. (a) Potential evolution during the stability test. (b) Polarization curves recorded between 0 mA · cm^{-2} and 50 mA · cm^{-2} at selected intervals during the stability test. (c) Nyquist plots (symbols) with corresponding equivalent-circuit fits (solid lines) recorded at selected intervals. (d) Bode plots (symbols) with corresponding fits (solid lines) recorded at selected intervals, with dashed lines indicating the frequency regions attributed to the adsorption related (R_a) and charge-transfer related (R_{ct}) processes. The fitted parameters R_{ct}, R_a, C_{dl}, and C_a are summarized in (e) and (f). The error bars represent the fitting uncertainty of the respective parameters obtained from the equivalent-circuit modelling.

Table S1: Selected PGM-free catalysts performances of published works in acidic media.

Catalyst	Overpotential @10mA · cm^{-2}	Stability	Notes	Reference
**$Cu_{1.5}Mn_{1.5}O_4$:F**	330 mV	24h @ 10 mA · cm^{-2}	Co-free	[1]
**Co_2MnO_4**	400 mV	1500h @ 200 mA · cm^{-2}	Benchmark for PGM-free	[2]
**γ-MnO_2**	490 mV	8000h @ 10 mA · cm^{-2}	Reference point for pure Mn oxide	[3]
**Li-Co_3O_4**	510 mV	16h @ 10 mA · cm^{-2}	Co-based	[4]
**α+γ-Fe_2O_3**	650 mV	24h @ 10 mA · cm^{-2}	Reference point	[5]
**$Co_{0.05}Fe_{0.95}O_y$**	650 mV	85h @ 10 mA · cm^{-2}	Reference point	[6]
**$Mn_{0.7}Sb_{0.3}O_y$**	670 mV	4k cycles	Reference point	[7]
**$Ni_{0.5}Mn_{0.5}Sb_{1.7}O_y$**	670 mV	168h @ 10 mA · cm^{-2}	Reference point	[8]
**$Fe_{1.5}Mn_{0.5}O_3$**	550 mV	120h @ 10 mA · cm^{-2}		[9]
**$Fe_{1.5}Mn_{0.5}O_3$**	530 mV	77h @ 50 mA · cm^{-2}		This work

Physiochemical characterization

DFT-calculated Raman spectroscopy

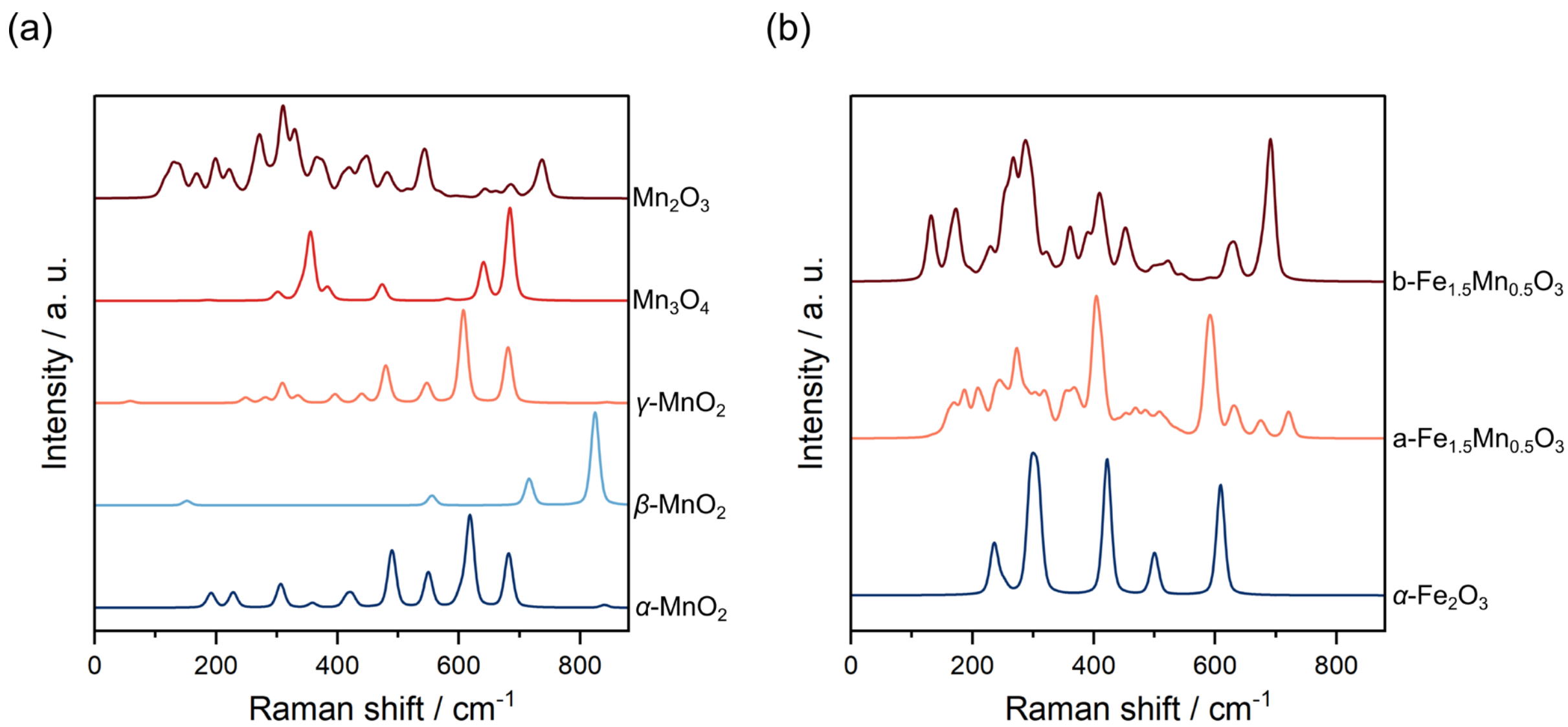

Figure S7: Density functional theory (DFT)-calculated Raman spectra between 0 and 880 cm^{-1} of (a) selected manganese oxide reference phases (α-MnO_2, β-MnO_2, γ-MnO_2, Mn_3O_4, and α-Mn_2O_3) and (b) hematite (α-Fe_2O_3) together with two structural models of Mn-substituted hematite (a-$Fe_{1.5}Mn_{0.5}O_3$ and b- $Fe_{1.5}Mn_{0.5}O_3$) used for comparison with the experimental Raman spectra.

Calculated Fe/Mn—O bond lengths

Table S2: Calculated Fe–O, and Mn–O bond lengths of the optimized crystal structures of α-Fe_2O_3 (*R-3c*), a-$Fe_{1.5}Mn_{0.5}O_3$ (*P1*) and b-$Fe_{1.5}Mn_{0.5}O_3$ (*P1*) obtained from the density functional theory (DFT) geometry optimization.

**α-Fe₂O₃** (*R-3c*)		**a-Fe1.5Mn0.5O3** (*P1*)		**b-Fe1.5Mn0.5O3** (*P1*)	
bond	Å	bond	Å	bond	Å
Fe1-O1	1.95	Fe1-O8	1.91	Fe1-O4	1.91
Fe1-O1	2.12	Fe1-O12	1.97	Fe1-O5	1.94
		Fe1-O9	2.01	Fe1-O1	1.98
		Fe1-O4	2.02	Fe1-O12	2.07
		Fe1-O5	2.13	Fe1-O7	2.17
		Fe1-O2	2.21	Fe1-O9	2.25
		Fe2-O7	1.91	Fe2-O3	1.91
		Fe2-O11	1.97	Fe2-O6	1.95
		Fe2-O3	2.01	Fe2-O2	1.97
		Fe2-O10	2.01	Fe2-O11	2.09
		Fe2-O6	2.13	Fe2-O8	2.16
		Fe2-O1	2.21	Fe2-O10	2.24
		Fe3-O3	1.91	Fe3-O12	1.93
		Fe3-O1	1.95	Fe3-O8	1.98

		Fe3-O5	1.98	Fe3-O9	1.98
		Fe3-O7	2.08	Fe3-O4	2.08
		Fe3-O12	2.17	Fe3-O5	2.11
		Fe3-O9	2.24	Fe3-O2	2.14
		Fe4-O4	1.91	Fe4-O11	1.92
		Fe4-O2	1.95	Fe4-O7	1.98
		Fe4-O6	1.97	Fe4-O10	1.99
		Fe4-O8	2.10	Fe4-O3	2.09
		Fe4-O11	2.16	Fe4-O6	2.12
		Fe4-O10	2.23	Fe4-O1	2.12
		Fe5-O7	1.93	Fe5-O11	1.91
		Fe5-O11	1.97	Fe5-O7	1.97
		Fe5-O9	1.98	Fe5-O9	2.00
		Fe5-O3	2.08	Fe5-O3	2.02
		Fe5-O1	2.12	Fe5-O1	2.13
		Fe5-O6	2.14	Fe5-O6	2.22
		Fe6-O8	1.92	Fe6-O12	1.91
		Fe6-O12	1.98	Fe6-O8	1.97
		Fe6-O10	1.99	Fe6-O10	2.01
		Fe6-O4	2.09	Fe6-O4	2.01
		Fe6-O2	2.12	Fe6-O2	2.13
		Fe6-O5	2.13	Fe6-O5	2.23
		Mn1-O5	1.92	Mn1-O1	1.92
		Mn1-O1	1.94	Mn1-O5	1.94
		Mn1-O9	1.97	Mn1-O9	1.97
		Mn1-O12	1.98	Mn1-O7	1.98
		Mn1-O4	2.12	Mn1-O3	2.12
		Mn1-O7	2.27	Mn1-O12	2.28
		Mn2-O6	1.91	Mn2-O2	1.91
		Mn2-O2	1.92	Mn2-O6	1.91
		Mn2-O10	1.95	Mn2-O10	1.95
		Mn2-O11	2.01	Mn2-O8	2.00
		Mn2-O3	2.16	Mn2-O4	2.16
		Mn2-O8	2.23	Mn2-O11	2.24

Experimental Raman spectroscopy

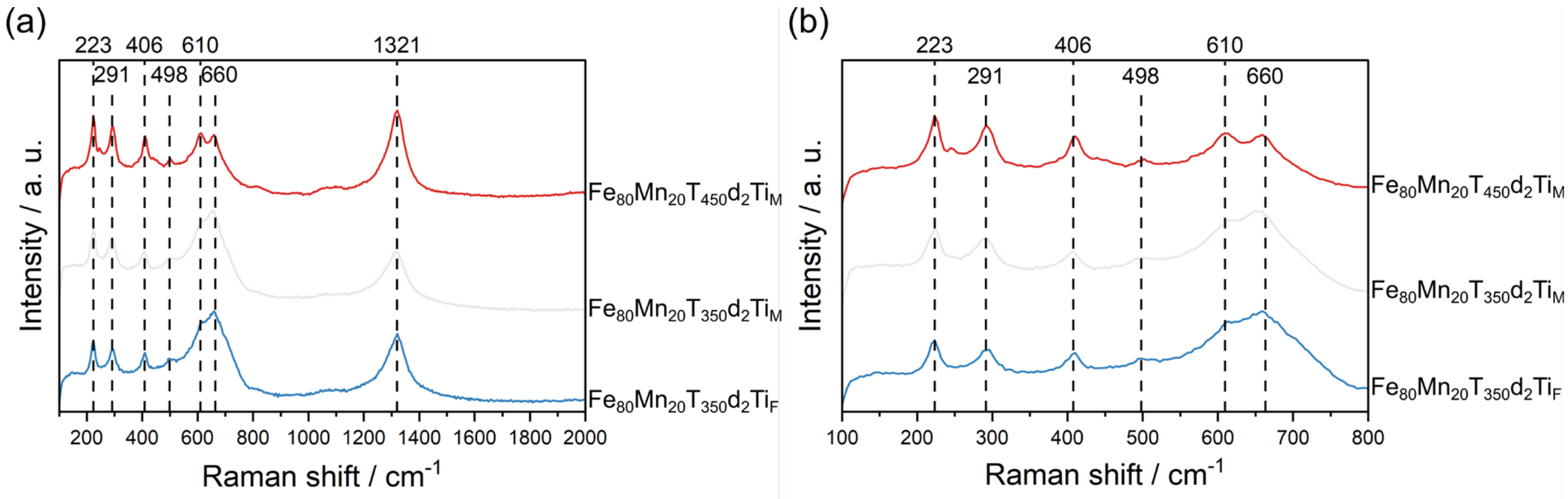

Figure S8: Experimental Raman spectra of $Fe_{80}Mn_{20}T_{350}d_2Ti_F$, $Fe_{80}Mn_{20}T_{350}d_2Ti_M$, and $Fe_{80}Mn_{20}T_{450}d_2Ti_M$ recorded using a 532 nm excitation wavelength and a laser power of 5%. The spectra are presented to compare the influence of the substrate type (the Ti foil and Ti mesh) and calcination temperature on the vibrational properties of the catalysts for (a) Raman shifts range of 100-2000 cm^{-1} and (b) 100-800 cm^{-1}.

Scanning electron microscope (SEM)

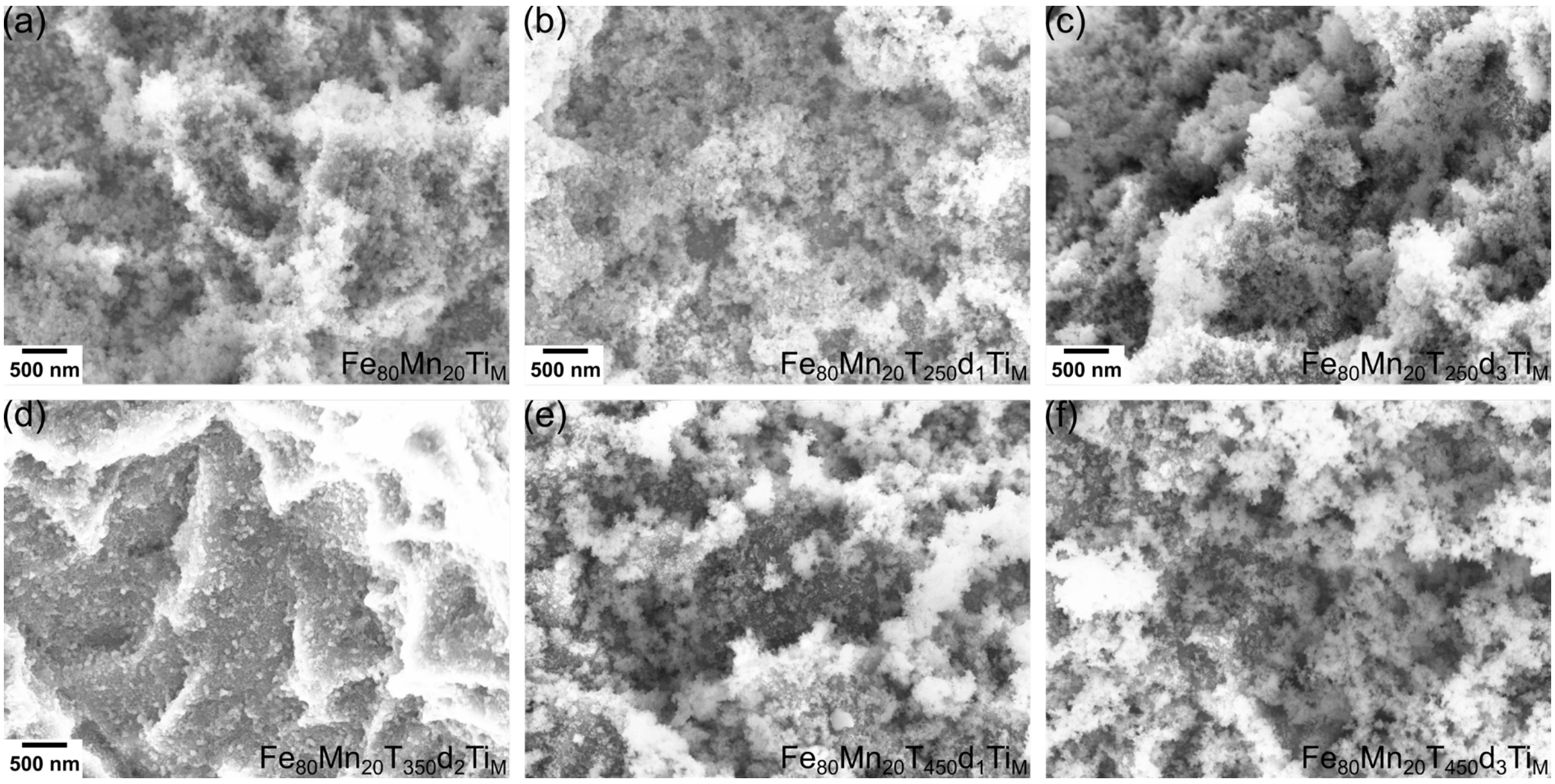

Figure S9: SEM images recorded at an accelerating voltage of 8 kV and a magnification of 20k for (a) uncalcined $Fe_{80}Mn_{20}Ti_M$, (b) $Fe_{80}Mn_{20}T_{250}d_1Ti_M$, (c) $Fe_{80}Mn_{20}T_{250}d_3Ti_M$, (d) $Fe_{80}Mn_{20}T_{350}d_2Ti_M$, (e) $Fe_{80}Mn_{20}T_{450}d_1Ti_M$, and (f) $Fe_{80}Mn_{20}T_{450}d_3Ti_M$, illustrating the influence of calcination temperature and duration on the surface morphology of the catalysts grown on the Ti mesh.

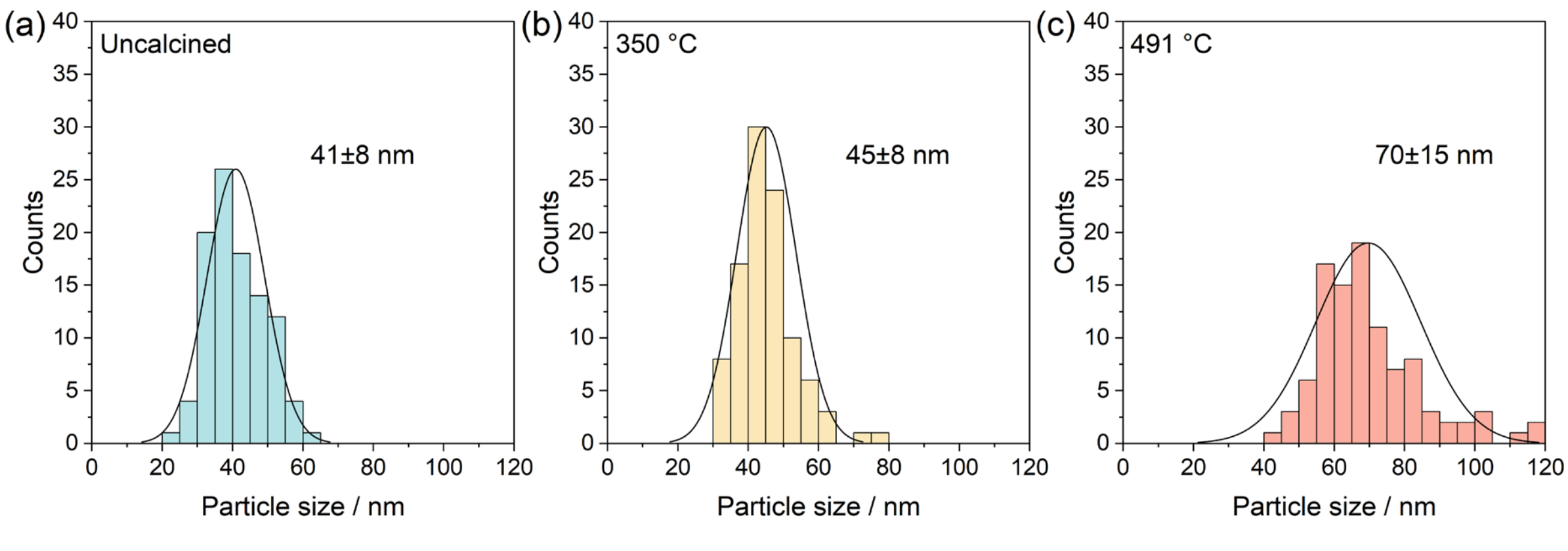

Figure S10. SEM-derived apparent particle size distributions obtained by measuring 100 particles for (a) an uncalcined electrode, (b) an electrode calcined at 350 °C, and (c) an electrode calcined at 491 °C.

Cross-sectional SEM

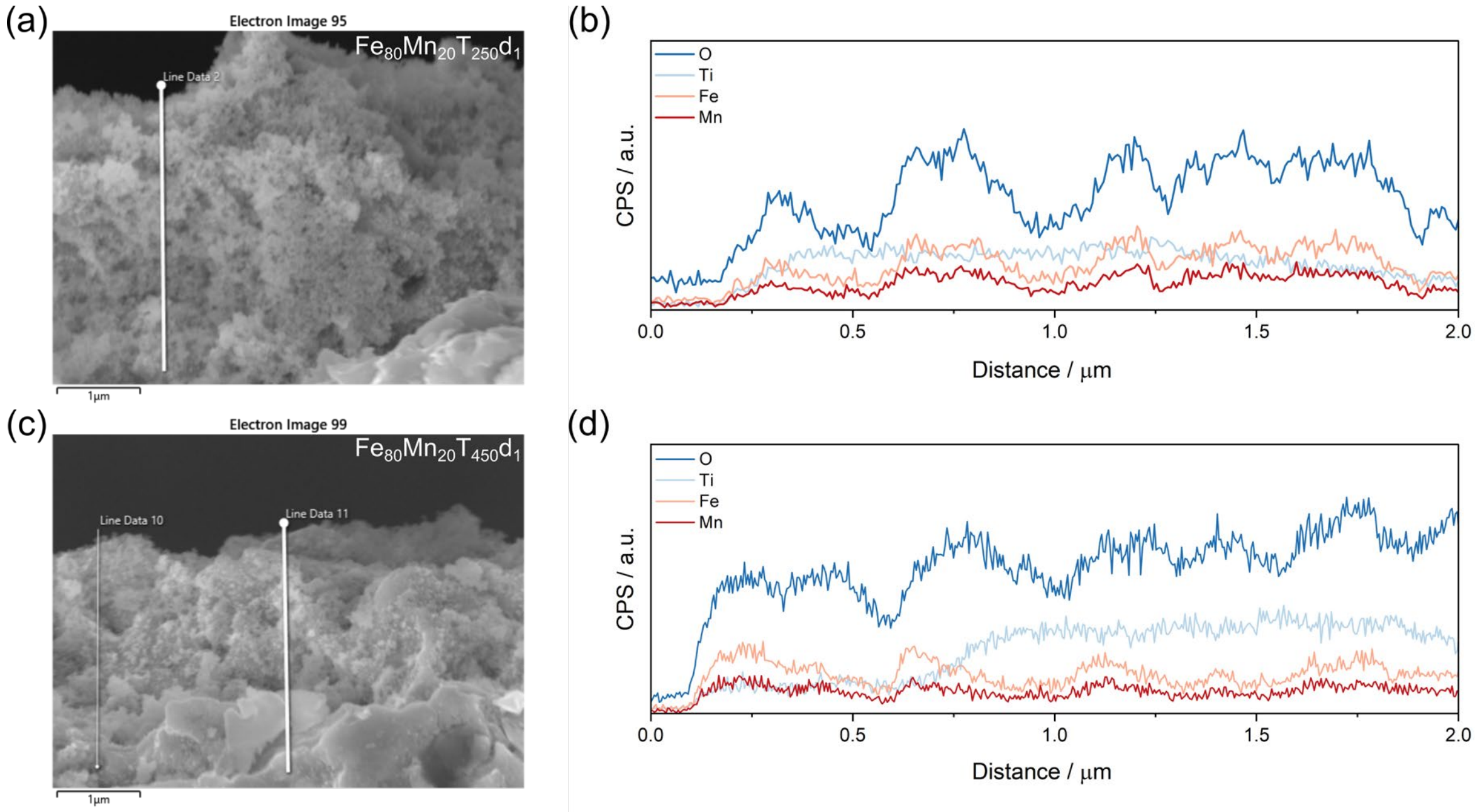

Figure S11: Cross-sectional SEM images recorded at an accelerating voltage of 8 kV for (a) $Fe_{80}Mn_{20}T_{250}d_1Ti_M$ and (c) $Fe_{80}Mn_{20}T_{450}d_1Ti_M$, together with the corresponding EDX line scans shown in (b) and (d), respectively. For both samples, line scans starting from the outer catalyst layer were recorded at multiple positions across the cross section, yielding comparable elemental distribution profiles and confirming a homogeneous metal distribution within the catalyst layers.

SEM – EDX

Table S3: SEM–EDX elemental compositions for the prepared catalysts deposited on the Ti mesh and Ti foil. Values are given in atomic percent, with standard deviations shown in parentheses.

Electrode	Fe content / at.-%	Mn content / at.-%	Fe:Mn
$Fe_{80}Mn_{20}Ti_M$ (uncalcined)	78.8 (1.2)	21.2 (1.2)	3.7
$Fe_{80}Mn_{20}Ti_M$ (uncalcined)	79.6 (0.7)	20.4 (0.7)	3.9
$Fe_{80}Mn_{20}T_{209}d_{2.0}Ti_M$	71.3 (1.0)	28.7 (1.0)	2.5
$Fe_{80}Mn_{20}T_{250}d_{1.0}Ti_M$	77.1 (3.3)	22.9 (3.3)	3.4
$Fe_{80}Mn_{20}T_{250}d_{3.0}Ti_M$	81.2 (2.6)	19.8 (2.6)	4.3
$Fe_{80}Mn_{20}T_{350}d_{0.6}Ti_M$	78.3 (1.7)	21.7 (1.7)	3.6
$Fe_{80}Mn_{20}T_{350}d_{2.0}Ti_M$	76.0 (3.6)	24.0 (3.7)	3.2
$Fe_{80}Mn_{20}T_{350}d_{2.0}Ti_M$	78.0 (2.4)	22.0 (2.5)	3.5
$Fe_{80}Mn_{20}T_{350}d_{3.4}Ti_M$	81.0 (1.3)	19.0 (1.6)	4.3
$Fe_{80}Mn_{20}T_{450}d_{1.0}Ti_M$	75.1 (4.1)	24.9 (4.3)	3.0
$Fe_{80}Mn_{20}T_{450}d_{3.0}Ti_M$	78.9 (2.1)	21.1 (2.2)	3.7
$Fe_{80}Mn_{20}T_{491}d_{2.0}Ti_M$	77.5 (0.9)	22.5 (1.0)	3.4
**Average**	**77.7 (1.1)**	**22.3 (1.2)**	**3.5 (0.5)**
**$Fe_{100}Mn_0T_{350}d_2Ti_F$**	100 (-)	0 (-)	-
**$Fe_{80}Mn_{20}Ti_F$ (uncalcined)**	76.8 (15.5)	23.2 (5.2)	3.3
**$Fe_{80}Mn_{20}Ti_F$ (uncalcined)**	78.7 (6.6)	21.3 (2.7)	3.7
**$Fe_{80}Mn_{20}T_{350}d_2Ti_F$**	78.1 (1.8)	21.9 (1.7)	3.6

Transmission electron microscope (TEM) – energy dispersive x-ray spectroscopy (EDX)

Table S4: TEM–EDX elemental compositions of the catalysts mechanically extracted from the Ti mesh electrodes. Values are given in atomic percent with standard deviations shown in parentheses.

Electrode	Fe content / at. %	Mn content / at.-%	Fe:Mn
$Fe_{100}Mn_0T_{350}d_2Ti_F$	99.8 (-)	0.2 (-)	-
$Fe_{80}Mn_{20}Ti_F$ (uncalcined)	76.8 (10.4)	23.2 (2.7)	3.3
$Fe_{80}Mn_{20}T_{350}d_2Ti_F$	45.9 (0.5)	54.1 (2.2)	0.9

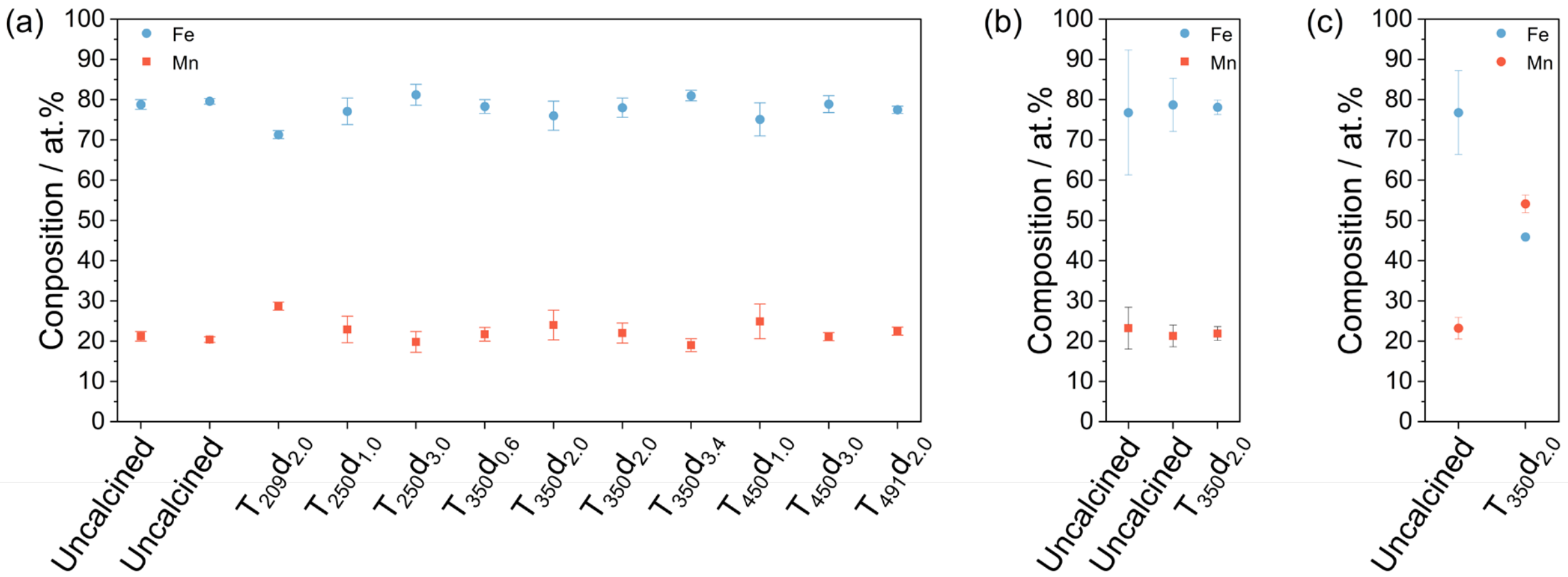

Figure S12: Comparison of semi-quantitative results by SEM-EDX at 8 kV accelerating voltage for (a) $Fe_{80}Mn_{20}$ grown on Ti mesh, and (b) $Fe_{80}Mn_{20}$ grown on Ti foil. Semi-quantitative results by TEM-EDX at 200 kV accelerating voltage for (c) $Fe_{80}Mn_{20}$ grown on Ti foil.

X-ray diffraction (XRD)

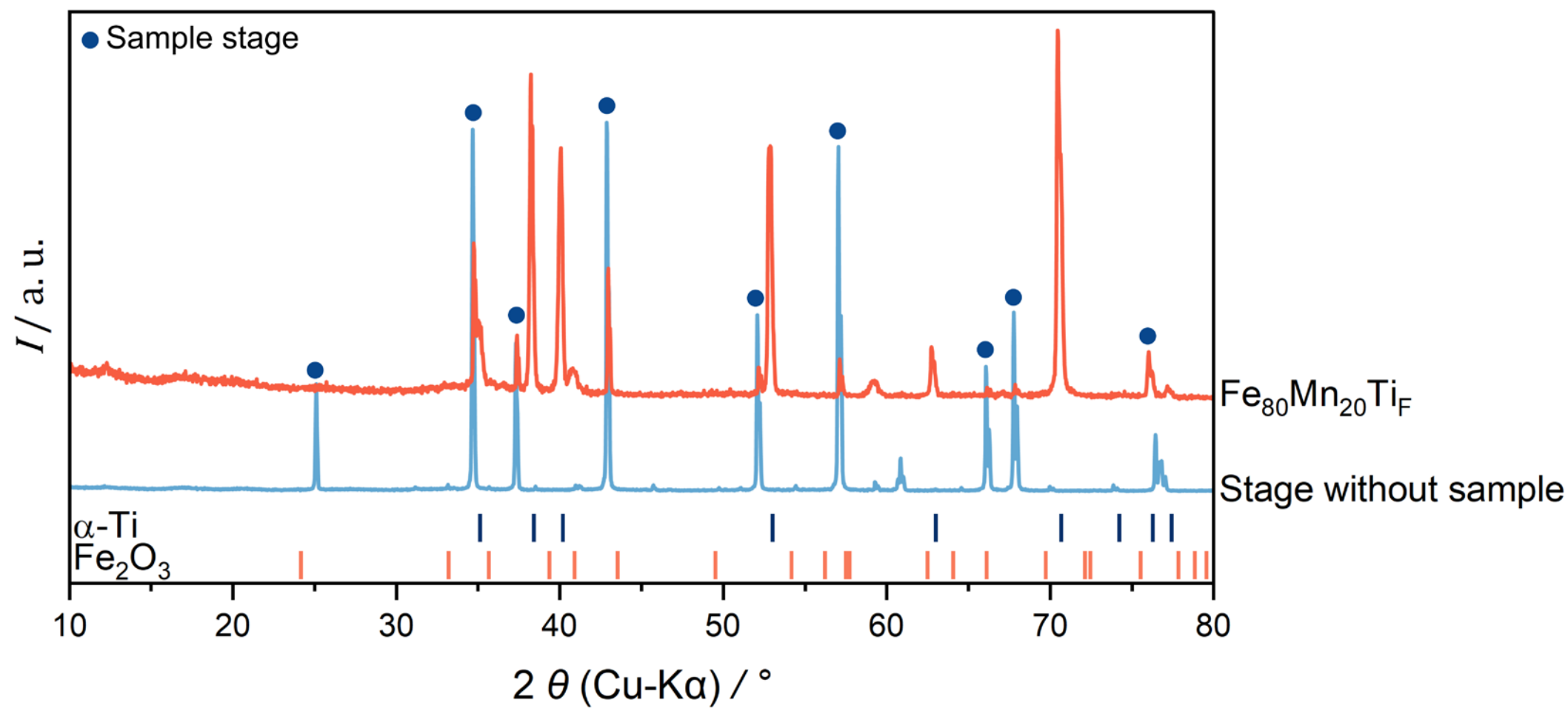

Figure S13: Comparison of the XRD patterns of uncalcined $Fe_{80}Mn_{20}Ti_F$ and the sample stage. The sharp reflections marked with ● originate from the sample stage and are not associated with the catalyst material. The same reflections were observed during the *in situ* HTXRD measurements of $Fe_{80}Mn_{20}Ti_F$, likely due to slight sample displacement or movement at the beginning of the measurement.

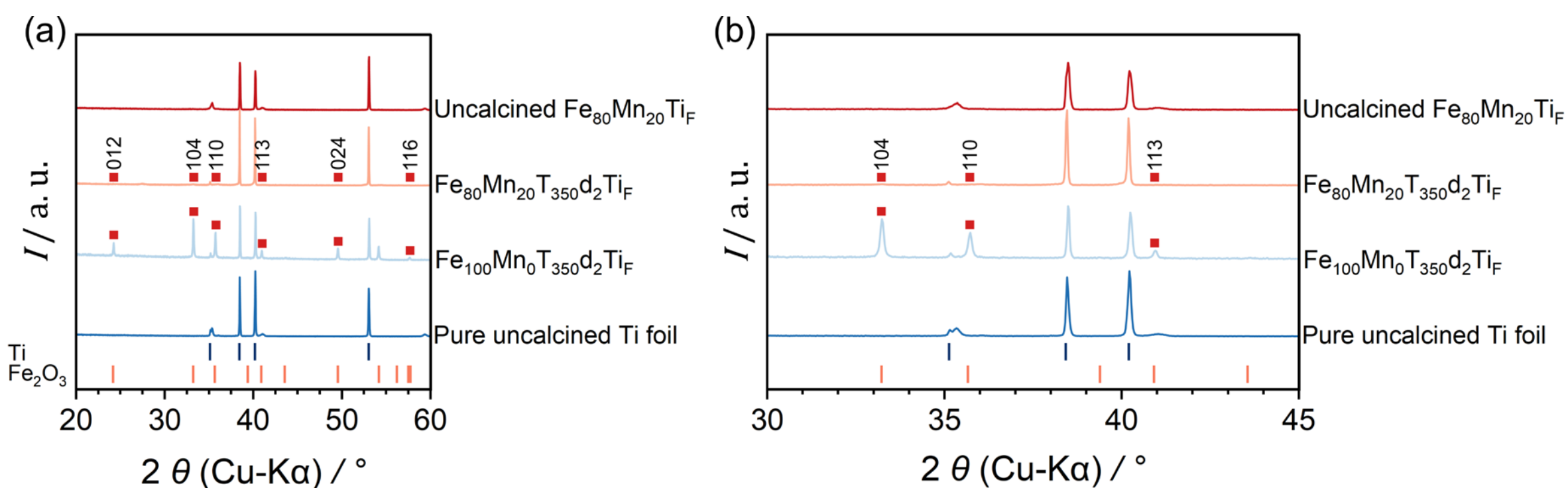

Figure S14: XRD patterns of uncalcined Ti foil, $Fe_{100}Mn_0T_{350}d_2Ti_F$, $Fe_{80}Mn_{20}T_{350}d_2Ti_F$, and uncalcined $Fe_{80}Mn_{20}Ti_F$: (a) 2θ range of 20–60° and (b) 2θ range of 30–45°.

In situ high-temperature XRD

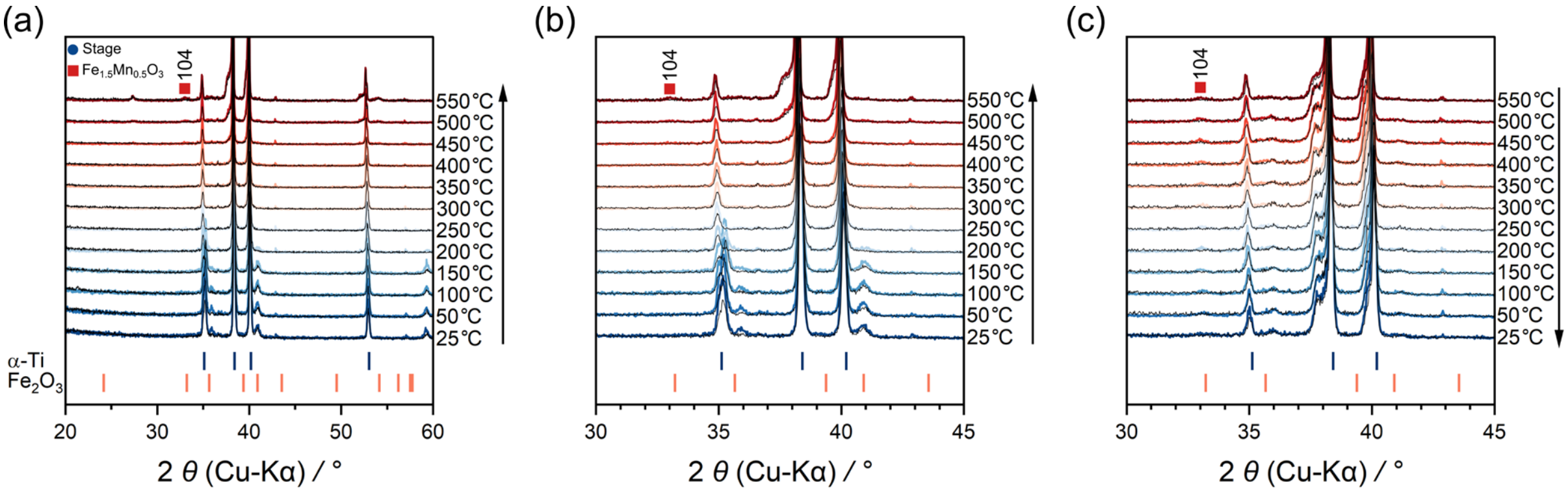

Figure S15: *In situ* HTXRD patterns of $Fe_{80}Mn_{20}Ti_M$ (colored) and the Ti mesh substrate (black) recorded in 50 °C increments during controlled heating: (a) 2θ = 20°-60° and (b) 2θ = 30°-45°. (c) Corresponding *in situ* HTXRD patterns recorded during controlled cooling in the range of $2\,\theta$ = 30°-45°. For clarity, only selected 2θ are shown.

The measurements indicate a similar formation of the hematite phase. However, the uneven surface of the Ti mesh leads to lower overall reflection intensities compared with the sample supported on the Ti foil. Consequently, only one reflection differs visibly between the pure Ti mesh and the foil (2θ ≈ 33°, corresponding to the main hematite peak). At temperatures above 500 °C, additional reflections appear (e.g., 2θ ≈ 27°, 37°, and 52°), likely to originate from the Ti mesh or the formation of TiO_2. As temperatures above 500 °C were not further investigated, this behavior is not discussed in detail.

Ex situ synchrotron wide angle X-ray scattering (WAXS)

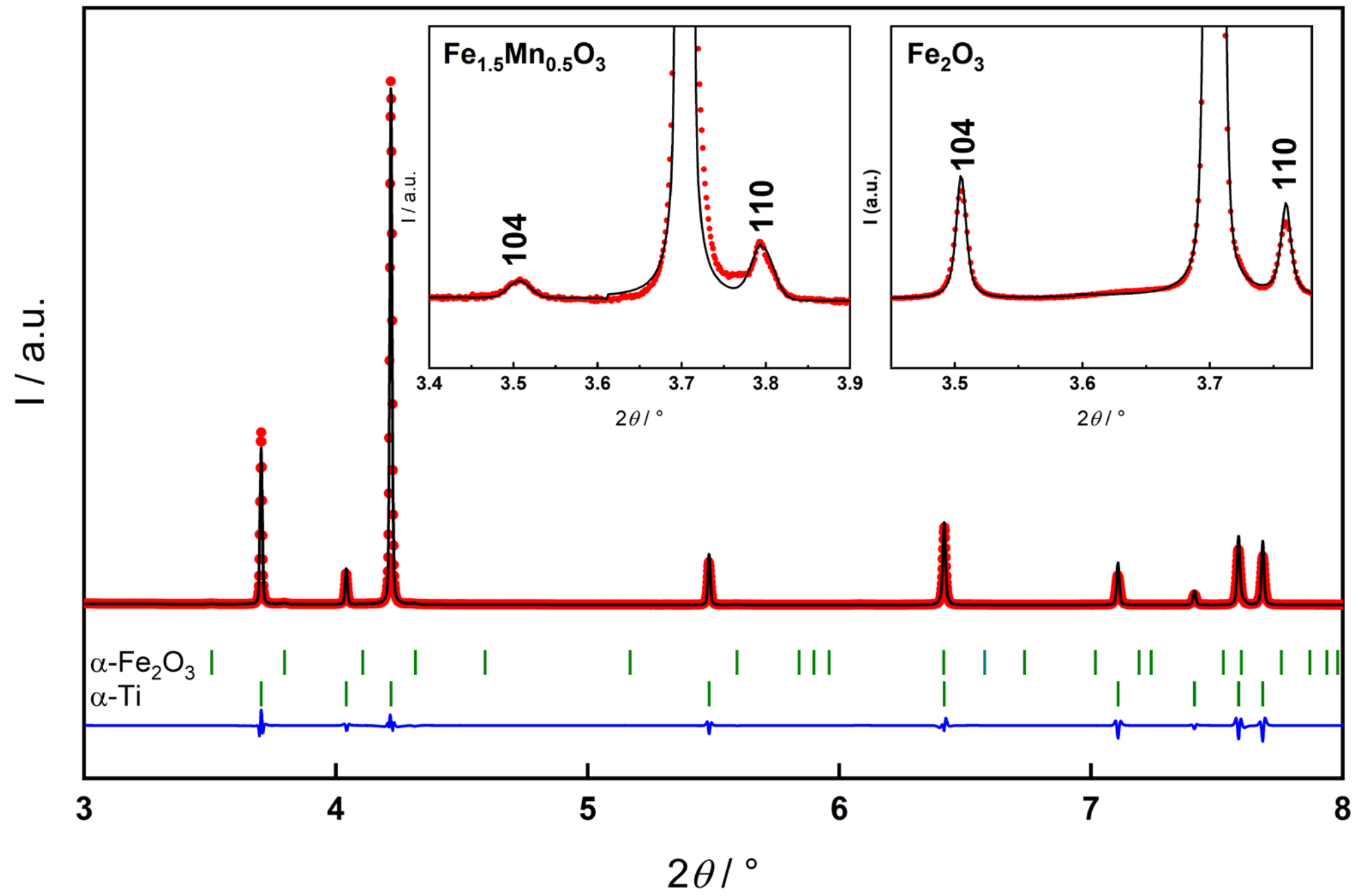

Figure S16: WAXS pattern (red data points) of $Fe_{100}Mn_0T_{350}d_2Ti_F$ (α-Fe_2O_3) and $Fe_{80}Mn_{20}T_{350}d_2Ti_F$ ($Fe_{1.5}Mn_{0.5}O_3$) grown on the Ti foil substrate, recorded using a high-energy synchrotron radiation with a wavelength of λ = 0.165 Å. The full diffraction pattern is shown for $Fe_{1.5}Mn_{0.5}O_3$ between 2θ = 3°-8°, while selected enlarged regions between 2θ = 3.4°-3.9° are displayed for both $Fe_{1.5}Mn_{0.5}O_3$ and α-Fe_2O_3. Le Bail-type profile fitting was performed using the FullProf software suite with α-Fe_2O_3 ($R\bar{3}c$) and α-Ti ($P6_3/mmc$) as reference crystal structures. The black and blue lines represent the fitted diffraction pattern and the difference between experimental and fitted profiles, respectively.

Thermogravimetric analyses (TGA), differential scanning calorimetry (DSC), and mass spectrometry (MS)

TGA-DSC-MS measurements were performed on the catalyst directly grown on the Ti foil. However, characteristic thermal and gas signals were not observed, likely due to the low material loading. Notably, the uncalcined powder sample showed no oxygen evolution reaction (OER) activity in 0.1 M $HClO_4$ when tested using a rotating disk electrode, indicating that significant support–catalyst interaction is required for activation. This powder-based measurement is included to support the analysis of hematite phase formation, complementing the *in situ* HTXRD results.

Post-stability characterization

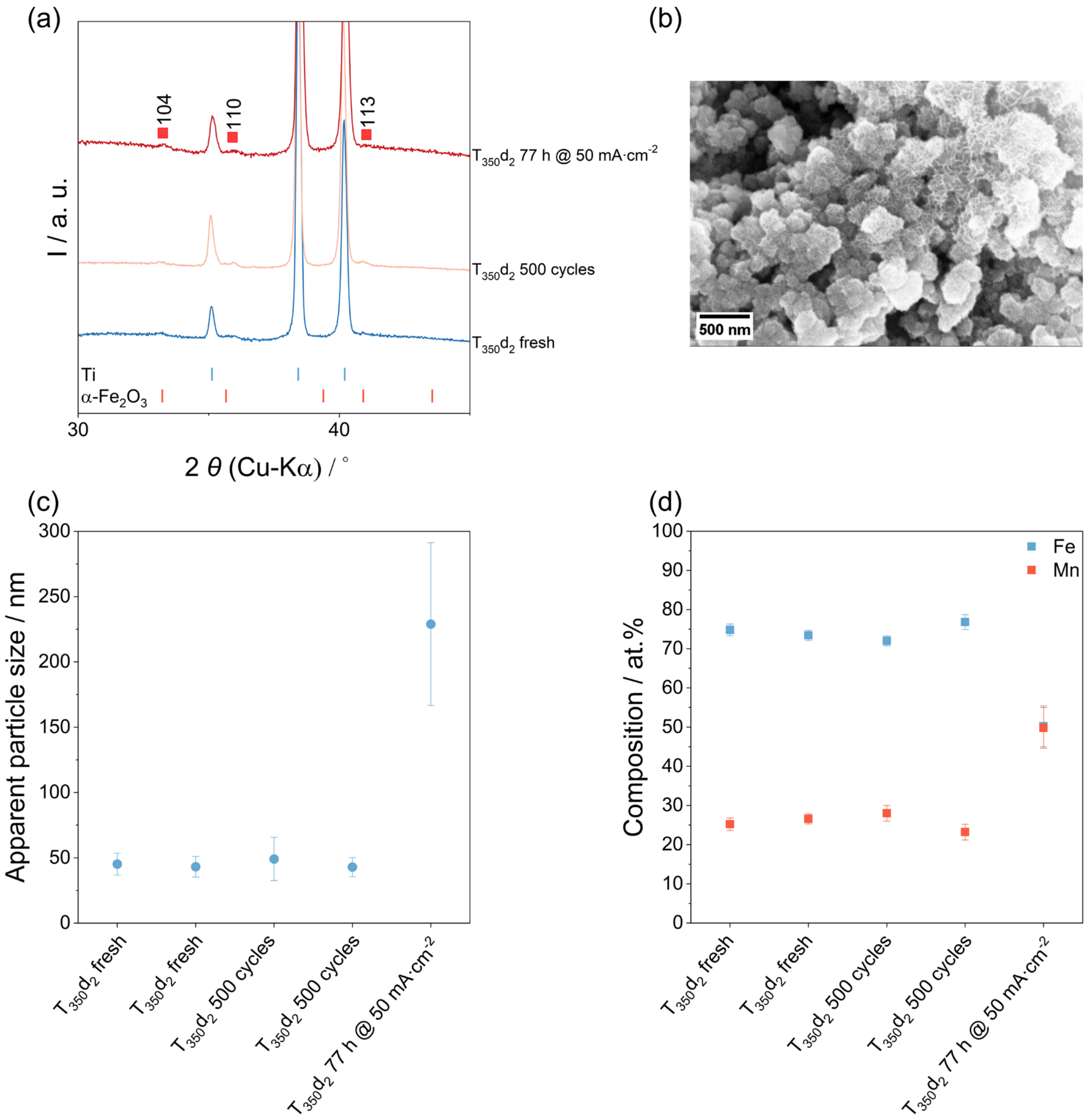

Figure S17: Post-stability characterization of the best-performing catalyst using fresh electrodes, electrodes subjected to 500 CV cycles, and an electrode subjected to extended durability testing at 50 mA · cm^{-2} for 77 h. (a) XRD patterns of the corresponding electrodes. (b) SEM-image of the electrode surface. (c) Apparent particle size distributions determined by SEM. (d) Semi-quantitative Fe and Mn compositions determined by SEM–EDX.

Mechanistic Insights into the OER from *operando* Raman

Electrochemical measurements for *operando* Raman spectroscopy

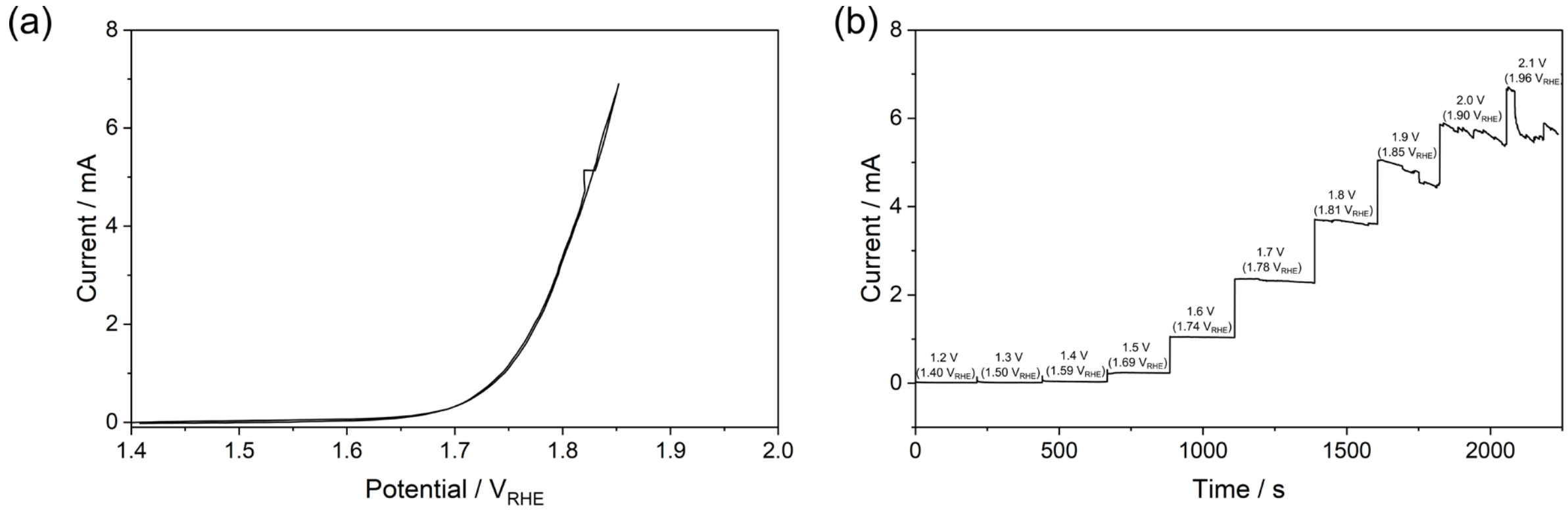

Figure S18: *Operando* Raman measurement for the $Fe_{1.5}Mn_{0.5}O_3$ grown on the Ti foil and calcined at 350 °C for 2 h, performed in a three-electrode setup with a 3 M KCl Ag/AgCl reference electrode and Pt wire counter electrode in 0.1 M $HClO_4$, which was continuously pumped into the cell. Electrochemical impedance spectroscopy (EIS) was conducted between 10 kHz and 1 Hz to ensure proper connection. Panel (a) shows the cyclic voltammogram (CV) between 1.4 V_{RHE} and 2.0 V_{RHE} at a scan rate of 10 mV · s^{-1}, while panel (b) shows the current recorded under constant potential application between 1.2 V (1.40 V_{RHE}) and 2.1 V (1.96 V_{RHE}).

Experimental section

Experimental conditions for the Design of experiments (DOE)

Table S5: Experimental conditions used for calcination optimization by the DOE following hydrothermal synthesis at 140 °C for 12 h on the Ti mesh. Catalysts grown on the Ti foil are also listed, with the letter "F" in the name indicating the use of the Ti foil instead of the Ti mesh.

Electrode	n($Fe(NO_3)_3$·9 H_2O) / mmol	n(KMnO4) / mmol	Calcination T / °C	Calcination t / h
$Fe_{80}Mn_{20}T_{209}d_2Ti_M$	8.0	2.0	209	2.0
$Fe_{80}Mn_{20}T_{491}d_2Ti_M$	8.0	2.0	491	2.0
$Fe_{80}Mn_{20}T_{250}d_1Ti_M$	8.0	2.0	250	1.0
$Fe_{80}Mn_{20}T_{350}d_{0.6}Ti_M$	8.0	2.0	350	0.6
$Fe_{80}Mn_{20}T_{450}d_1Ti_M$	8.0	2.0	450	1.0
$Fe_{80}Mn_{20}T_{450}d_3Ti_M$	8.0	2.0	450	3.0
$Fe_{80}Mn_{20}T_{350}d_{3.4}Ti_M$	8.0	2.0	350	3.4
$Fe_{80}Mn_{20}T_{350}d_2Ti_M$	8.0	2.0	350	2.0
$Fe_{80}Mn_{20}T_{250}d_3Ti_M$	8.0	2.0	250	3.0
$Fe_{80}Mn_{20}T_{350}d_2Ti_M$	8.0	2.0	350	2.0
$Fe_0Mn_0Ti_F$	0	0	350	2.0
**$Fe_{100}Mn_0T_{350}d_2Ti_F$**	4.0	0.0	350	2.0
**$Fe_{80}Mn_{20}T_{350}d_2Ti_F$**	3.2	0.8	350	2.0
**$Fe_{80}Mn_{20}Ti_F$ (uncalcined)**	3.2	0.8	-	-

Quantum chemical calculations

Monkhorst-Pack *k*-meshes

a-$Fe_{1.5}Mn_{0.5}O_3$: 6 × 6 × 4

b-$Fe_{1.5}Mn_{0.5}O_3$: 6 × 6 × 4

The optimized crystal structures and the *k*-meshes employed for Fe_2O_3 and the Mn oxides investigated are detailed in Ref. 3.

Calculated magnetic moments

Table S6: Calculated magnetic moments of *d*-metal atoms corresponding to spin populations.

**Fe_2O_3**		**a-$Fe_{1.5}Mn_{0.5}O_3$**		**b-$Fe_{1.5}Mn_{0.5}O_3$**	
atom	μ_B	atom	μ_B	atom	μ_B
Fe1	4.24	Mn1	3.92	Fe1	4.29
Fe2	4.24	Mn2	3.93	Fe2	-4.27
Fe3	-4.24	Fe1	4.30	Fe3	4.32
Fe4	-4.24	Fe2	-4.29	Fe4	-4.26
		Fe3	4.32	Mn1	3.95
		Fe4	-4.26	Mn2	3.93
		Fe5	4.33	Fe5	4.28
		Fe6	-4.26	Fe6	-4.28

Optimized structures in CIF format

a-$Fe_{1.5}Mn_{0.5}O_3$

```
data_findsym-output
_audit_creation_method FINDSYM

_cell_length_a    5.0697050500
_cell_length_b    5.3488736400
_cell_length_c    8.8262864300
_cell_angle_alpha 74.6920620000
_cell_angle_beta  87.8570320000
_cell_angle_gamma 62.8362640000
_cell_volume      204.4246703247

_symmetry_space_group_name_H-M "P 1"
_symmetry_Int_Tables_number 1
_space_group.reference_setting '001:P 1'
_space_group.transform_Pp_abc a,b,c;0,0,0

loop_
_space_group_symop_id
_space_group_symop_operation_xyz
1 x,y,z

loop_
_atom_site_label
_atom_site_type_symbol
_atom_site_symmetry_multiplicity
_atom_site_Wyckoff_label
_atom_site_fract_x
_atom_site_fract_y
_atom_site_fract_z
_atom_site_occupancy
_atom_site_fract_symmform

Mn1 Mn   1 a 0.47205  0.05575  0.82086  1.00000 Dx,Dy,Dz
Mn2 Mn   1 a -0.02671 0.05277  0.32509  1.00000 Dx,Dy,Dz
Fe1 Fe   1 a 0.04148  -0.06428 0.67638  1.00000 Dx,Dy,Dz
Fe2 Fe   1 a 0.54145  -0.06511 0.17629  1.00000 Dx,Dy,Dz
Fe3 Fe   1 a 0.79394  0.42462  -0.06882 1.00000 Dx,Dy,Dz
Fe4 Fe   1 a 0.29342  0.42310  0.43022  1.00000 Dx,Dy,Dz
Fe5 Fe   1 a 0.21532  0.56843  0.07246  1.00000 Dx,Dy,Dz
Fe6 Fe   1 a 0.71426  0.56933  0.57255  1.00000 Dx,Dy,Dz
O1  O    1 a 0.22489  0.25080  -0.03153 1.00000 Dx,Dy,Dz
O2  O    1 a 0.72578  0.24863  0.46923  1.00000 Dx,Dy,Dz
O3  O    1 a 0.66040  0.25594  0.11672  1.00000 Dx,Dy,Dz
O4  O    1 a 0.16312  0.25678  0.61778  1.00000 Dx,Dy,Dz
O5  O    1 a 0.71348  0.25233  0.77700  1.00000 Dx,Dy,Dz
O6  O    1 a 0.21247  0.24964  0.27777  1.00000 Dx,Dy,Dz
O7  O    1 a 0.78899  0.73027  0.03671  1.00000 Dx,Dy,Dz
O8  O    1 a 0.28949  0.73298  0.53539  1.00000 Dx,Dy,Dz
O9  O    1 a 0.31720  0.76979  0.87448  1.00000 Dx,Dy,Dz
O10 O    1 a 0.81932  0.76868  0.37422  1.00000 Dx,Dy,Dz
O11 O    1 a 0.27084  0.75803  0.22389  1.00000 Dx,Dy,Dz
O12 O    1 a 0.76880  0.76153  0.72333  1.00000 Dx,Dy,Dz

# end of cif
```

b-$Fe_{1.5}Mn_{0.5}O_3$

```
data_findsym-output
_audit_creation_method FINDSYM

_cell_length_a    5.0726127400
_cell_length_b    5.3469589155
_cell_length_c    8.8225245200
_cell_angle_alpha 74.7425272006
_cell_angle_beta  87.7904940000
_cell_angle_gamma 62.8490879861
_cell_volume      204.4918147984

_symmetry_space_group_name_H-M "P 1"
_symmetry_Int_Tables_number 1
_space_group.reference_setting '001:P 1'
_space_group.transform_Pp_abc a,b,c;0,0,0

loop_
_space_group_symop_id
_space_group_symop_operation_xyz
1 x,y,z

loop_
_atom_site_label
_atom_site_type_symbol
_atom_site_symmetry_multiplicity
_atom_site_Wyckoff_label
_atom_site_fract_x
_atom_site_fract_y
_atom_site_fract_z
_atom_site_occupancy
_atom_site_fract_symmform

Fe1 Fe   1 a 0.54439  -0.07594 0.18148  1.00000 Dx,Dy,Dz
Fe2 Fe   1 a 0.04398  -0.07715 0.68026  1.00000 Dx,Dy,Dz
Fe3 Fe   1 a -0.03500 0.06834  0.32240  1.00000 Dx,Dy,Dz
Fe4 Fe   1 a 0.46448  0.06875  0.82263  1.00000 Dx,Dy,Dz
Mn1 Mn   1 a 0.22217  0.55588  0.07071  1.00000 Dx,Dy,Dz
Mn2 Mn   1 a 0.72293  0.55301  0.57502  1.00000 Dx,Dy,Dz
Fe5 Fe   1 a 0.79262  0.43451  -0.07290 1.00000 Dx,Dy,Dz
Fe6 Fe   1 a 0.29212  0.43399  0.42706  1.00000 Dx,Dy,Dz
O1  O    1 a 0.46391  0.75240  0.02699  1.00000 Dx,Dy,Dz
O2  O    1 a -0.03751 0.74984  0.52763  1.00000 Dx,Dy,Dz
O3  O    1 a -0.08687 0.75639  0.86760  1.00000 Dx,Dy,Dz
O4  O    1 a 0.41015  0.75572  0.36659  1.00000 Dx,Dy,Dz
O5  O    1 a -0.02565 0.75153  0.21790  1.00000 Dx,Dy,Dz
O6  O    1 a 0.47520  0.74984  0.71845  1.00000 Dx,Dy,Dz
O7  O    1 a 0.51844  0.26238  -0.02695 1.00000 Dx,Dy,Dz
O8  O    1 a 0.02030  0.25865  0.47378  1.00000 Dx,Dy,Dz
O9  O    1 a 0.06696  0.27004  0.12450  1.00000 Dx,Dy,Dz
O10 O    1 a 0.56859  0.26930  0.62427  1.00000 Dx,Dy,Dz
O11 O    1 a 0.03976  0.23231  0.78566  1.00000 Dx,Dy,Dz
O12 O    1 a 0.53906  0.23017  0.28690  1.00000 Dx,Dy,Dz

# end of cif
```

References

[1] Patel, P. P.; Datta, M. K.; Velikokhatnyi, O. I.; et al. "Noble metal-free bifunctional oxygen evolution and oxygen reduction acidic media electro-catalysts." *Scientific Reports* **2016**, 28367, https://doi.org/10.1038/srep28367.

[2] Li, A., Kong, S.; Guo, C.; et al. "Enhancing the stability of cobalt spinel oxide towards sustainable oxygen evolution in acid." *Nature Catalysis* **2022**, 5.2, 109-118, https://doi.org/10.1038/s41929-021-00732-9.

[3] Li, A.; Ooka, H.; Bonnet N.; et al. "Stable potential windows for long-term electrocatalysis by manganese oxides under acidic conditions." *Angewandte Chemie International Edition* **2019**, 58.15, 5054-5058, https://doi.org/10.1002/anie.201813361.

[4] Etzi Coller Pascuzzi, M.; van Velzen, M.; Hofmann, J.P.; Hensen, J. M. "On the stability of Co_3O_4 oxygen evolution electrocatalysts in acid." *ChemCatChem* **2021**, 13.1, 459-467, https://doi.org/10.1002/cctc.202001428.

[5] Kwong, W. L.; Lee, C. C.; Shchukarev, A.; Björn, E.; Messinger, J. "High-Performance Iron (**III**) Oxide Electrocatalyst for Water Oxidation in Strongly Acidic Media." *Journal of Catalysis* **2018**, *365*, 29-35, https://doi.org/10.1016/j.jcat.2018.06.018.

[6] Kwong, W. L.; Lee, C. C.; Shchukarev, A.; Björn, E.; Messinger, J. "Cobalt-doped hematite thin films for electrocatalytic water oxidation in highly acidic media." *Chemical Communications* **2019**, 55.34, 5017-5020, https://doi.org/10.1039/c9cc01369e.

[7] Zhou, L.; Shinde, A.; Montoya, J.H.; et al. "Rutile alloys in the Mn–Sb–O system stabilize Mn^{3+} to enable oxygen evolution in strong acid." *ACS Catalysis* **2018**, 8.12, 10938-10948, https://doi.org/10.1021/acscatal.8b02689.

[8] Moreno-Hernandez, I. A.; MacFarland, C. A.; Read, C. G., Papadantonakis K. M.; Brunschwig, B. S.; Lewis, N. S. "Crystalline nickel manganese antimonate as a stable water-oxidation catalyst in aqueous 1.0 M H_2SO_4." *Energy & Environmental Science* **2017**, 10.10, 2103-2108, https://doi.org/10.1039/c7ee01486d.

[9] Rosenthal, M. K.; Moumaneix, L.; Rautama, E.-L.; et al. "Optimizing an iron-and manganese-based electrocatalyst for the oxygen evolution reaction in a proton exchange membrane electrolyzer." *Chemical Engineering Journal* **2025**, 172461, https://doi.org/10.1016/j.cej.2025.172461.

[10] Shrestha, N. K.; Patil, S. A.; Han, J.; et al. "Chemical etching induced microporous nickel backbones decorated with metallic Fe@ hydroxide nanocatalysts: an efficient and sustainable OER anode toward industrial alkaline water-splitting." *Journal of Materials Chemistry* **2022***, A 10 (16)*, 8989-9000, https://doi.org/10.1039/d1ta10103j.

[11] Kuklin, M. S.; Eklund, K.; Linnera, J.; Ropponen, A.; Tolvanen, N.; Karttunen, A. J. "Structural properties and magnetic ground states of 100 binary *d*-metal oxides studied by hybrid density functional methods" *Molecules* **2022***, 27(3),* 874, https://doi.org/10.3390/molecules27030874.
